# Experimentally controlled downregulation of the histone chaperone FACT in *Plasmodium berghei* reveals that it is critical to male gamete fertility

**DOI:** 10.1111/j.1462-5822.2011.01683.x

**Published:** 2011-12

**Authors:** Eliane C Laurentino, Sonya Taylor, Gunnar R Mair, Edwin Lasonder, Richard Bartfai, Hendrik G Stunnenberg, Hans Kroeze, Jai Ramesar, Blandine Franke-Fayard, Shahid M Khan, Chris J Janse, Andrew P Waters

**Affiliations:** 1 Leiden Malaria Research Group, Department of Parasitology, Leiden University MedicalAlbinusdreef 2, 2333 ZA Leiden, the Netherlands; 2 Institute of, Infection, Immunity & Inflammation, School of Medical, Veterinary & Life Sciences, & Wellcome Centre for Molecular Parasitology, Glasgow Biomedical Research Centre, University of GlasgowScotland, UK; 3 Molecular Parasitology Unit, Instituto de Medicina MolecularLisbon, Portugal; 4 Centre for Molecular and Biomolecular Informatics, NCMLS, Radboud University Nijmegen Medical CentreNijmegen, the Netherlands; 5 Department of Molecular Biology, NCMLS, University of NijmegenNijmegen, the Netherlands

## Abstract

Human FACT (facilitates chromatin transcription) consists of the proteins SPT16 and SSRP1 and acts as a histone chaperone in the (dis)assembly of nucleosome (and thereby chromatin) structure during transcription and DNA replication. We identified a *Plasmodium berghei* protein, termed FACT-L, with homology to the SPT16 subunit of FACT. Epitope tagging of FACT-L showed nuclear localization with high expression in the nuclei of (activated) male gametocytes. The gene encoding FACT-L could not be deleted indicating an essential role during blood-stage development. Using a ‘promoter-swap’ approach whereby the fact-l promoter was replaced by an ‘asexual blood stage-specific’ promoter that is silent in gametocytes, transcription of fact-l in promoter-swap mutant gametocytes was downregulated compared with wild-type gametocytes. These mutant male gametocytes showed delayed DNA replication and gamete formation. Male gamete fertility was strongly reduced while female gamete fertility was unaffected; residual ookinetes generated oocysts that arrested early in development and failed to enter sporogony. Therefore FACT is critically involved in the formation of fertile male gametes and parasite transmission. ‘Promoter swapping’ is a powerful approach for the functional analysis of proteins in gametocytes (and beyond) that are essential during asexual blood-stage development.

## Introduction

Nuclear DNA is tightly packaged into chromatin which comprises of histones, non-histone proteins and RNAs which influences processes like transcription, replication, repair and recombination. For the cellular machinery to access the DNA chromatin must be unwound and the DNA temporarily cleared of histone proteins. Conversely, the DNA has to be repackaged into chromatin afterwards. Histone chaperones play an essential role in assembling and disassembling chromatin and the ordered formation of the nucleosome structure (Ransom *et al*., 2010). Nucleosomes are modular assemblies of stable heterodimers of histones H2A/H2B and H3/H4 associated with 146 bp of DNA. Histones are highly basic proteins and require chaperones for proper interaction in the formation of chromatin. Such proteins include nucleosome assembly protein 1 (NAP-1), nucleoplasmin, Asf1 (antisilencing function), CAF-1 (chromatin assembly factor), FACT (facilitates chromatin transcription) and HirA (histone regulator A) (Eitoku *et al*., 2008; Ransom *et al*., 2010).

The life cycle of malaria parasites consists of an alternation between periods of rapid multiplication requiring extensive DNA replication and periods of cellular differentiation, each phase requiring transcription of different sets of stage-specific genes (for review see Kooij *et al*., 2006).*Plasmodium* chromatin must therefore continuously exist as a dynamic entity, switching between the open and closed forms at specific nuclear regions and loci based on the requirements of the cell. While *Plasmodium* contains homologues of all the assembly/disassembly factors mentioned above (except nucleoplasmin) to date just two nucleosome assembly proteins (NAPs) have been characterized in *Plasmodium*, termed NapL and NapS (Navadgi *et al*., 2006; Gill *et al*., 2009). NapL interacts with both core and linker histones in the cytoplasm and transfers the cytoplasmic histones on to NapS which is localized in the nucleus and which deposits histones H3–H4 and H2A and H2B onto DNA (Navadgi *et al*., 2006; Gill *et al*., 2010). Both NAP proteins are likely essential during asexual blood-stage development as targeted deletion of their genes was unsuccessful (Gill *et al*., 2009; 2010). It has been shown that NapS is highly expressed in the mature male gametocyte (Khan *et al*., 2005) where it accumulates in the nucleus (Pace *et al*., 2006).

FACT is a histone chaperone that has received considerable attention for its function in facilitating RNA transcription and DNA replication by dynamically altering chromatin through modifications of nucleosome structure (Xin *et al*., 2009; Ransom *et al*., 2010). FACT is an evolutionarily conserved, abundant nuclear heterodimer/trimer. The large subunit FACT-L, in humans p140h/SPT16, is a homologue of the yeast Spt16/Cdc68 protein (suppressor of Ty). The small subunit of FACT (FACT-S) in human structure-specific recognition protein 1 (SSRP1), possesses a C-terminal high-mobility group (HMG) box with an N-terminus homologous to the yeast protein Pob3 (DNA polymerase one binding). In yeast SSRP1 activity is represented by two distinct proteins, Pob3 and Nhp6A (or B) which encodes the missing HMG box.

Functionally, both yeast and human FACT act during transcription initiation and displace histones H2A/H2B to effect nucleosome disassembly and facilitate transcription elongation (Formosa *et al*., 2002; Belotserkovskaya *et al*., 2003; Biswas *et al*., 2005).

During DNA replication (Wittmeyer and Formosa, 1997; Okuhara *et al*., 1999; Schlesinger and Formosa, 2000; Tan *et al*., 2006), human and mouse FACT localize to replication origins (Hertel *et al*., 1999; Tan *et al*., 2006) and yeast FACT co-purified as part of the replication fork progression complex (Gambus *et al*., 2006). A role for human FACT in chromatin disassembly during DNA replication has also been shown by its ability to promote replication initiation *in vivo* and to promote DNA unwinding by the MCM helicase on nucleosome templates *in vitro* (Tan *et al*., 2006). The FACT complex plays an essential role in cells as its depletion affects cell viability through defects in DNA replication (Okuhara *et al*., 1999).

The genome of the human parasite *Plasmodium falciparum* encodes a gene (PFE0870w) with clear homology to FACT-L that contains the peptidase-M24, Spt16 and Rtt106 domains of SPT16 (GeneDB: PFE0870w). Attempts to disrupt fact-l in the rodent malaria parasite *Plasmodium berghei* were unsuccessful (RMgm-255) highlighting an essential role in asexual blood-stage development. *Plasmodium* FACT-L is, like the nucleosome chaperone NapS, abundantly expressed in male gametocytes of *P. berghei* (Khan *et al*., 2005). The abundance of the histone chaperone FACT in male gametocytes indicates active processes of nucleosome assembly and disassembly that may be required for gene expression and/or for DNA replication during the rapid generation of eight haploid gametes by a single, male gametocyte. In order to analyse the function of FACT specifically in gametocytes we have developed a ‘promoter-swap’ approach to downregulate transcription of fact-l in *P. berghei* gametocytes while not affecting expression in the asexual blood-stage parasites. Male gametocytes lacking fact-l (Δfact-l_gam_ gametes) show delayed gamete formation and strongly reduced fertility. The abundant presence of FACT-L in the nucleus of mature male gametocytes during DNA replication and the absence of a phenotype in female gametocytes lacking transcription of fact-l indicates that FACT in mature gametocytes is critically involved in the process of the formation of fertile male gametes.

## Results

### Identification of *Plasmodium* FACT

**Fig. 1:**
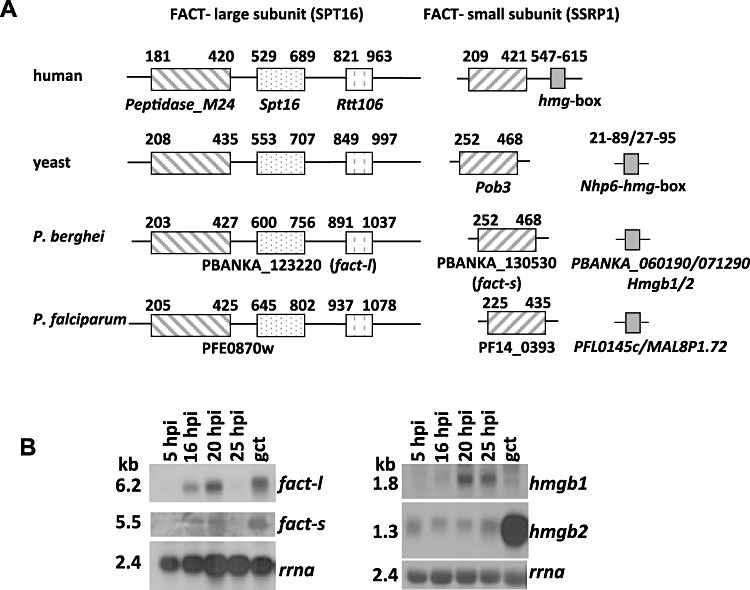
Characterization of *P. berghei* proteins of the FACT complex. A. Schematic representation of proteins of the FACT complex of human, yeast and *Plasmodium* showing the (location of) conserved domains (see Figs S2 and S3 for the alignment of the gene sequences). The human small unit protein, ssrp1, contains a high-mobility group (hmg) box domain which is absent in the small unit of yeast (Pob3) and *Plasmodium* and small genes that encode HMG boxes are illustrated for both yeast (nhp6a or b) and *Plasmodium* (hmg2a or b). B. Northern analyses of transcripts of the large subunit, *fact-l* and the small subunit facts (left panel) and the two *Plasmodium* genes encoding HMG-box containing proteins, *hmgb1* and *hmgb2* (right panel). Transcripts were analysed in synchronized blood stages at different hours post infection (hpi) (5 hpi: ring forms; 16 hpi: mature trophozoites, 20 hpi: immature schizonts; 25 hpi: mature schizonts; gct: purified gametocytes).

In a proteome analysis of purified male and female *P. berghei* gametocytes a protein was identified that appeared highly abundant in males (Fig. S1; Khan *et al*., 2005) but was absent in females. blast-based homology searches with PBANKA_123220 identified clear homology to the eukaryotic SPT16 protein (e.g. 30% identity, 41% similarity to SPT16 of *Saccharomyces cerevisiae*) with an identical arrangement of the Rttp106-like, SPT16 and M24 peptidase domains; we refer to this *Plasmodium* SPT16 homologue as FACT-L (Fig. S2; GeneDB, http://www.genedb.org). FACT-S (*P. berghei*: PBANKA_130530; *P. falciparum*: PF14_0393) is also abundantly expressed in male gametocytes (Fig. S1; Khan *et al*., 2005) and is highly homologous to the N-termini of huSSRP1 and yeast Pob3 (30% identity, 54% similarity, Figs 1 and S3). *S. cerevisiae* FACT-S/Pob3 lacks the HMG box present at the C-terminus of several vertebrate members, which instead is encoded by two separate and redundant genes, nhp1a and b. *P. berghei* FACT-S also lacks the HMG box and two other *Plasmodium* proteins have been previously identified as potential NHP6 homologues that contain a closely related class II HMG box (Fig. S4): they are HMGB1 (PFL0145c; PBANKA_060190) with 55% identity (65% similarity) to *S. cerevisiae* NHP6A (Briquet *et al*., 2006) and HMGB2 (MAL8P1.72; PBANKA_071290) which has 52% identity, 63% similarity to *S. cerevisiae* NHP6A (Kumar *et al*., 2008). In summary, like yeast FACT the *P. berghei* FACT complex is a likely heterotrimer consisting of FACT-L, FACT-S and HMGB1 (or 2).

Transcriptome and proteome analyses of various *Plasmodium* species have provided solid evidence for expression of all FACT subunits in blood-stage parasites (http://www.PlasmoDB.org). Here we determined by Northern analysis the transcription patterns of each individual FACT component during a synchronized *P. berghei* blood-stage infection. In asexual-stage parasites *fact-l*, *fact-s* and *hmgb1* show similar transcription patterns with a clearly visible transcript in mature trophozoites [16 hpi (hours post infection)] and a peak in dividing schizonts (20 hpi), while in rings and mature schizonts transcription is low or absent (Fig. 1). Transcription of *hmgb1* appeared absent in gametocytes, whereas *fact-l*, *fact-s* and *hmgb2* were strongly transcribed (Fig. 1B). Our results are similar to the transcription patterns identified in other *Plasmodium* species (Briquet *et al*., 2006; Gissot *et al*., 2008). Despite the strong Northern signal for *hmgb2*, HMGB2 was not detected by mass-spectrometry in *P. berghei* gametocyte proteomes consistent with the prediction that it is translationally repressed (Mair *et al*., 2006). HMGB1 on the other hand was present at low levels in both male and female gametocytes [three peptides total (Hall *et al*., 2005; Khan *et al*., 2005)].

### Expression of *P. berghei* FACT-L in blood-stage parasites

**Fig. 2:**
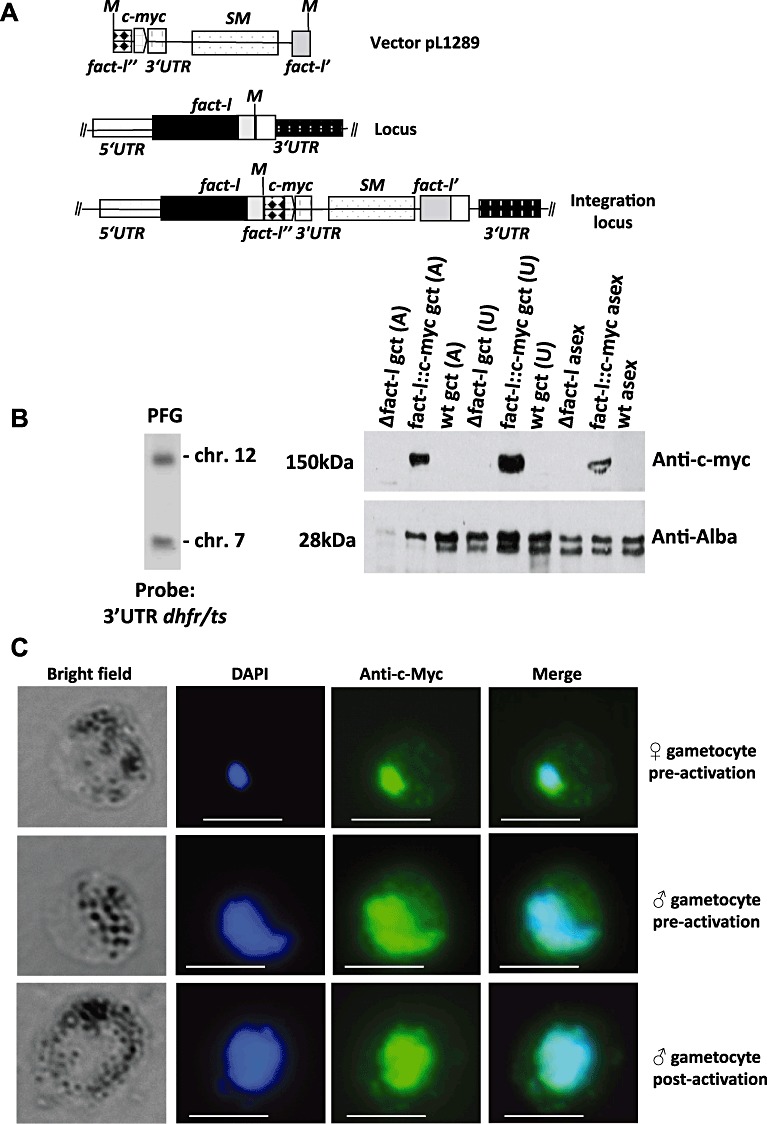
Epitope tagging of FACT-L to demonstrate subcellular localization. A. Schematic representation of the linearized vector pL1289 for C-terminal epitope tagging of *fact-l* with *c-myc* by integration through single-cross-over homologous recombination. The *fact-l* locus is shown before and after the integration event. The integration results in a c-myc-tagged version of fact-l and an incomplete fact-l copy (SM = *tgdhfr-ts* selectable marker; M = Mscl; fact-l' and ” indicate the C-terminal encoding fragments of fact-l after linearization in the tagging vector). B. Left panel: Southern analysis of separated chromosomes of fact-l::c-myc parasites showing correct integration of the c-myc-tagging vector pL1289 (see A). Hybridization with the 3′-*dhfr/ts* probe recognizes the construct integrated into *fact-l* on chromosome 12 and the endogenous dhft/ts gene on chromosome 7. Right panel: Western analysis of expression of c-Myc-tagged FACT-L in blood stages of fact-l::c-myc parasites as shown by reaction with anti-c-Myc antibodies. Rabbit polyclonal peptide antibody Alba-3 is used as a loading control. Activated gametocytes (A), Unactivated gametocytes (U). PFG: pulse field gel; Asex: mixed asexual blood stages; gct: gametocytes. C. The subcellular localization of Fact-L is shown by immunofluorescence analysis of male and female gametocytes of fact-l::c-myc parasites using anti-c-Myc antibodies. FACT-L (green) colocalizes with the DAPI-stained nuclei (blue) of male (flattened, peripherally distributed nucleus) and female (compact nucleus) gametocytes (pre- and post-activation). Scale bars = 6 µm.

Generation of anti-peptide antibodies specific for FACT-L was unsuccessful (data not shown). Therefore, FACT-L was epitope-tagged at its C-terminus and a cloned parasite line (1054cl1; fact-l::c-myc) generated (Fig. 2A) that showed no changes during asexual blood-stage development and presented wild-type ookinete formation. Western analyses of protein extracts from transgenic gametocytes and asexual blood stages using anti-c-Myc antibodies confirmed the expression of FACT-L in intraerythrocytic parasites (Fig. 2B). The cellular localization of FACT-L in blood stages of fact-l::c-myc was determined by IFA-analysis using anti-c-Myc antibodies (Fig. 2C). Immunostaining was strong in females and strongest in the large, eccentrically located nuclei of male gametocytes. After activation of male gametocytes, during the process of genome replication, the clear signal persisted throughout the entire, enlarged nucleus (Fig. 2C). In nuclei of asexual blood-stage parasites the fluorescent signal was considerably weaker with leakage into the cytoplasm (Fig. S5A); control IFAs with the anti-c-Myc antibody were therefore carried out using wild-type parasites. These experiments revealed the same background staining; particularly in schizonts the apical end was prominently highlighted while in other stages staining was significantly lower than the signal observed for the fact-l::c-myc parasites (Fig. S5B). We conclude that FACT-L is located predominantly in the parasite nucleus with evidence that a small proportion of FACT-L is located in the cytoplasm.

### FACT-L co-precipitates with FACT-S and DNA-binding proteins in gametocytes

In order to identify proteins that interact with FACT-L, immunoprecipitation (IP) experiments using gametocyte extracts from transgenic parasites expressing FACT-l::c-Myc were performed. Mass-spectrometric (LC-MS/MS) analysis of IP eluates resulted in the identification of 16 proteins with FACT-S as the second most abundant protein (Fig. S1). Neither HMGB1/2 nor any additional known nucleosome components or chromatin remodelling factors were identified in these IPs, but 10 RNA-binding proteins (including seven small and two large ribosomal subunit proteins) co-precipitated that were more abundant in female than male gametocytes (Khan *et al*., 2005). Two proteins relatively more abundant in male gametocytes co-purified with FACT both of which contain ALBA domains which are highly conserved modulators of chromatin structure (Fig. S1).

### Knock-down of fact-l transcription in gametocytes

**Fig. 3:**
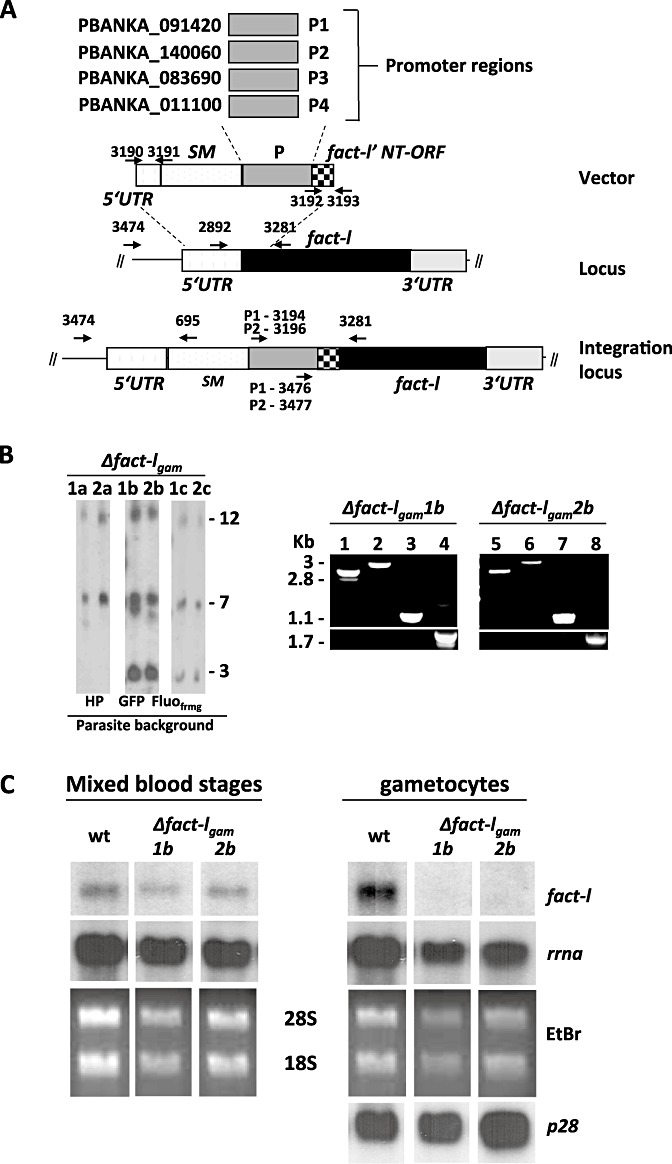
Development of a ‘promoter-swap’ technology for replacing the *fact-l* promoter with an ‘asexual-specific’ promoter. A. Schematic representation of the promoter-swap vector and the promoter regions of four selected genes, P1–P4. This vector integrates into the *fact-l* locus through a double-cross-over integration event resulting in replacement of part of the *fact-l* promoter region with the *tgdhfr-ts* selectable marker (SM) and one of the P1-P4 promoters. The location of primers for diagnostic PCRs (see B) for correct integration is shown. Fact-l' NT-ORF represents the N-terminal encoding region of fact-l used in the vector to ensure correct integration. B. Left panel: Southern analysis of separated chromosomes of six independent Δfact-l_gam_ mutants showing correct integration of the promoter-swap vectors containing promoter P1 (pL1312; 1a–c) or promoter P2 (pL1313; 2a–c). Hybridization with the 3′-*dhfr/ts* probe recognizes the construct integrated into the fact-l locus on chromosome 12, the endogenous *dhft/ts* gene on chromosome 7 and the transgenes of the parent parasite lines wt_GFP_ and wt-Fluo_frmg_ integrated into chromosome 3 (GFP in 1b, 2b and GFP/RFP in 1c, 2c). Upper panels: Diagnostic PCR analysis showing correct integration of vectors containing promoter P1 and P2 in Δfact-l_gam_ 1b and Δfact-l_gam_ 2b respectively (see A and Table S1 for primer location and sequence). Lanes 1, 5: primers 3474/695; lane 2, 3: promoter P1, primers 3194/3281 and primers 3476/3281; promoter P2, lane 6: primers 3196/3281; lane 7: primers 3477/3281. Amplification of the wild-type FACT promoter region is negative in Δfact-l_gam_ 1b and Δfact-l_gam_ 2b lanes 4, 8 respectively using primers 2892/3281. Lower panels: Amplifications using the same primer pairs indicated for the upper panels with wt PB ANKA DNA as template. C. Northern analyses of *fact-l* transcription in wild type and two Δfact-l_gam_ mutants, showing the presence of the two Δfact-l_gam_ mutants in mixed blood stages but the absence of *fact-l* transcripts in purified Δfact-l_gam_ gametocytes; hybridization of *p28* is included to indicate similar amount of gametocyte RNA loading.

The disruption of fact-l using the standard approach for targeted gene deletion using constructs designed to integrate by double-cross-over homologous recombination (Menard and Janse, 1997) was unsuccessful in three independent experiments using two different targeting constructs (pL1230 and pL1147). This failure indicates that FACT-L is almost certainly essential for asexual blood-stage development (RMgm-255 for further details). To analyse the role of FACT-L in sexual-stage parasites we developed a ‘promoter-swap’ approach to completely eliminate or significantly reduce fact-l transcription in gametocytes. We generated several DNA constructs in order to replace the endogenous fact-l promoter (Fig. 3A) with one from a gene that was expected to be silent in gametocytes but would retain wild-type fact-l transcript (and thus protein) levels in asexual blood stages (see Fig. 1B).

Four ‘asexual blood stage-specific’ promoter regions of 2 kb were selected based on transcription data of genes during synchronized *P. berghei* blood-stage development (Hall *et al*., 2005) and comparison with *P. falciparum* transcription and proteome profiles (http://www.PlasmoDB.org). These genes were: P1: PBANKA_091420; (PB000331.03.0; conserved *Plasmodium* protein, unknown function), P2: PBANKA_140060 (PB000405.02.0; cytoadherence linked asexual protein, putative), P3: PBANKA_083690 (PB200042.00.0; BIR protein) and P4: PBANKA_011100; PB000528.00.0; 6-cysteine protein; P12). Transfection with the four ‘promoter-swap constructs’ resulted in the selection of pyrimethamine-resistant parasite populations with constructs P1 and P2 in two independent transfections for each construct (P1: Δfact-l_gam_1a, 1044 in cl15cy1; Δfact-l_gam_ 1b, 1076cl1, cl3 in 507cl2) (P2: Δfact-l_gam_ 2a, 1045cl1,cl2 in cl15cy1; Δfact-l_gam_ 2b, 1076cl1,cl2 in 507cl2). Transfections with constructs P3 and P4 did not generate recombinant parasites possibly due to insufficient production of FACT in blood-stage asexual parasites. In addition we generated ‘promoter-swap mutants’ in the Fluo-frmg reference transgenic line of *P. berghei* ANKA (RMgm164) which expresses GFP and red fluorescent protein (RFP) under the control of a male- and female-specific promoter respectively (P1: Δfact-l_gam_ 1c, 1134cl1,cl2; P2: Δfact-l_gam_ 2c, 1135cl1,cl2). Correct integration of constructs P1 and P2 were confirmed by Southern analyses of separated chromosomes and diagnostic PCR analyses (Fig. 3B). When we analysed transcription of fact-l by Northern blot, transcripts were present in asexual blood stages of Δfact-l_gam_ 1b and Δfact-l_gam_ 2b but absent in gametocytes (Fig. 3C). For further phenotype analyses we used the following cloned lines: Δfact-l_gam_ 1b, Δfact-l_gam_ 2b, Δfact-l_gam_ 1c and Δfact-l_gam_ 2c.

Male gametes of Δfact-l_gam_ show strongly reduced fertility. In standard synchronized infections all four promoter-swap mutants showed normal gametocyte production with 16–23% of ring forms developing into mature gametocytes (data not shown) which is comparable to gametocyte production in wild-type parasites (van Dijk *et al*., 2010). In addition, gamete formation in Δfact-l_gam_ parasites as determined by counting exflagellation and free female gametes in preparations at 20 min after activation of gamete formation was similar to that observed in wild-type parasites; i.e. more than 90% of the Δfact-l_gam_ gametocytes developed into gametes as determined by counting exflagellating male gametocytes and female gametes escaped from their host erythrocyte in Giemsa-stained smears 20 min after activation (data not shown).

**Fig. 4:**
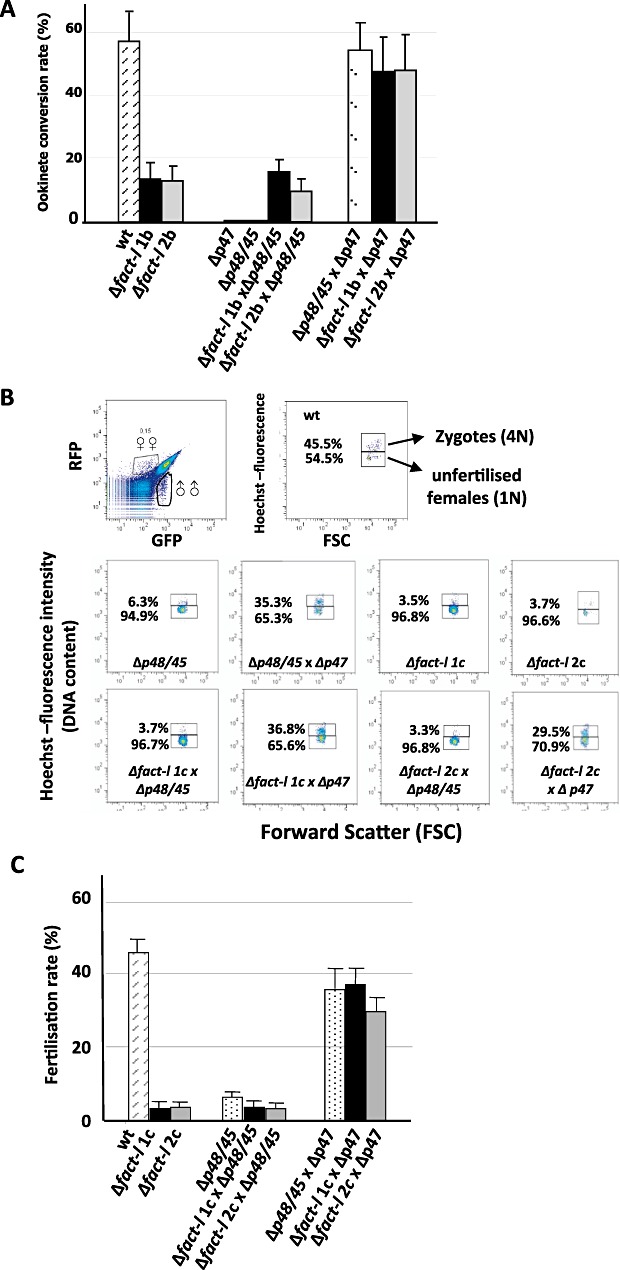
Fertility of Δfact-l_gam_ male gametes is strongly reduced. A. Fertility of Δfact-l_gam_ gametocytes as measured by the capacity to form ookinetes *in vitro* and by crossing Δfact-l_gam_ gametes with fertile female gametes of ΔP48/45 and fertile males of ΔP47. Δfact-l_gam_ gametocytes show strongly reduced capacity to form ookinetes. Δfact-l_gam_ female gametes are fertilized by fertile ΔP47 male gametes at wild-type levels, whereas Δfact-l_gam_ males show a strongly reduced capacity to form ookinetes after fertilization with the fertile ΔP48/45 females. The observations shown are from three independent experiments each performed in triplicate. The Δfact-l 1b and 2b mutant lines are in a GFP+ background. B. Fertility of Δfact-l_gam_ gametes as measured by the capacity to form 4N zygotes *in vitro* by FACS analysis of the fluorescence intensity (DNA content) of Hoechst-33258-stained, unfertilized female gametes (1N) and zygotes (4N) in the Fluofrmg background, which generates GFP+ve male gametocytes and RFP+ve female gametocytes. Female gametes and zygotes were selected for analysis of DNA content on basis of their RFP expression as shown in the upper, left dot plot. DNA content is measured 4 h after activation when meiosis should be completed in Hoechst-stained cells and the fertilization rate calculated (upper right dot plot). Analyses of cultures of Δfact-l_gam_ gametocytes show a strongly reduced capacity to form zygotes (middle panel right dot plots). C. Crossing of Δfact-l_gam_ gametes with fertile female gametes of ΔP48/45 and fertile males of ΔP47 (lower panel) show a strongly reduced capacity of male Δfact-l_gam_ gametes to produce zygotes after fertilization with the fertile female gametes of ΔP48/45. The graph shows the mean fertilization rates and standard deviations, defined as the percentage of females that develop into zygotes, as determined in triplicate in three independent crossing experiments. These Δfact-l 1c and 2c mutant lines express GFP and RFP in males and females respectively.

The fertility of the Δfact-l_gam_ gametes was initially analysed using standard assays for *in vitro* ookinete development. Δfact-l_gam_ parasites showed strongly reduced ability for ookinete formation *in vitro* with a reduction of more than 75% in Δfact-l_gam_ 1b and Δfact-l_gam_ 2b (Fig. 4A) (as determined by counting GFP+ free female gametes and GFP+ ookinetes). As shown for other mutants, such ookinete formation defects result from lower fertilization rates (caused by infertile male or female gametes; e.g. van Dijk *et al*., 2001) or abortion of zygote to ookinete transformation (Khan *et al*., 2005; Reininger *et al*., 2005; 2009; Tewari *et al*., 2005; Mair *et al*., 2006; 2010). Sex-specific fertility defects can be determined by *in vitro* cross-fertilization studies, where mutant gametes are cross-fertilized with gametes of parasite lines that produce either only fertile male or female gametes (Mair *et al*., 2010; van Dijk *et al*., 2010). Therefore, mixed gametes of Δfact-l_gam_ 1b or Δfact-l_gam_ 2b were crossed with either fertile Δp48/45 females (males are infertile) or fertile Δp47 male gametes (females are infertile). Δfact-l_gam_ males cross-fertilized with fertile Δp48/45 females showed no change in ookinete numbers formed when compared with pure mutant cultures. In contrast, Δfact-l_gam_ female gametes, when fertilized with fertile Δp47 males, showed normal rates of ookinete development. These results indicate that the fertility of male Δfact-l_gam_ gametes was strongly reduced, whereas that of females was normal.

To analyse in greater detail possible fertilization and meiosis defects we generated two additional promoter-swap mutants (Δfact-l_gam_ 1c, Δfact-l_gam_ 2c; see above) in the reporter line RMgm164; this line has RFP expression exclusive to female gametocytes that persists throughout ookinete development and therefore permits identification of female gametes and zygotes for FACS analysis of their DNA contents by Hoechst staining (Ponzi *et al*., 2009; Mair *et al*., 2010). Such analyses made 4 h after activation, when the zygote normally has completed meiosis, reveal cell ploidy and are therefore a quantitative indicator of fertilization success and early zygote development from the diploid to the tetraploid state (Fig. 4B). Here, these studies show very low levels of fertilization and meiosis in the Δfact-l_gam_ mutants with a reduction of more than 75% compared with wild type. In addition these studies confirmed normal fertilization rates and meiosis in Δfact-l_gam_ female gametes when fertilized with fertile Δp47 males, whereas fertile Δp48/45 females showed strongly reduced fertilization and meiosis when cross-fertilized with the Δfact-l_gam_ male gametes (Fig. 4B and C).

### Delayed DNA replication and male gamete formation by Δfact-l_gam_ gametocytes

**Fig. 5:**
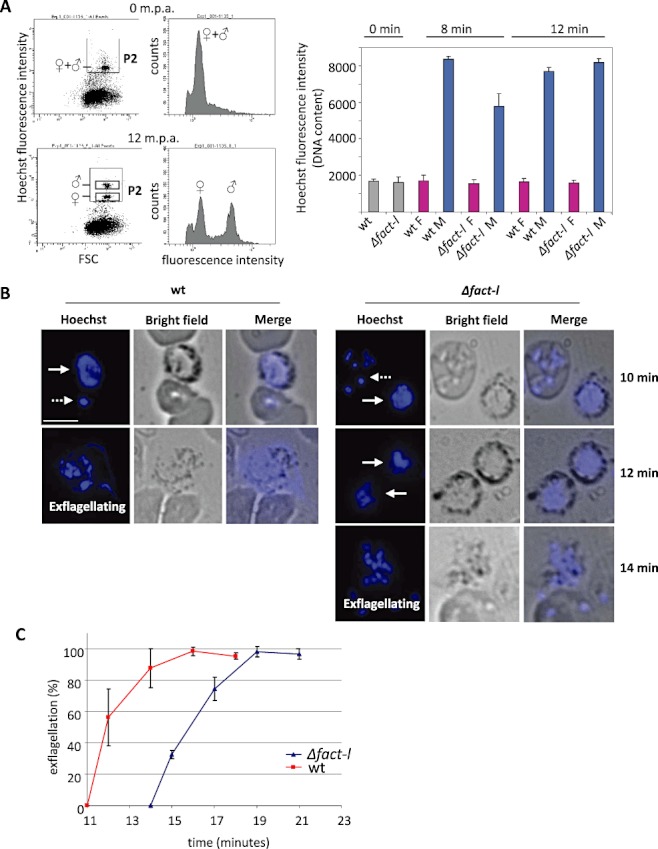
Male Δfact-l_gam_ gametocytes show delayed DNA replication and formation of gametes. A. Left panels: Determination of DNA content of male and female gametocytes at start of and 12 min post activation (m.p.a.). DNA content was determined in glutaraldehyde fixed and Hoechst-33258-stained purified gametocytes. Before activation (0 m.p.a.) males and females show the same low DNA content (upper FACS dot plot and graph). Ten to 12 m.p.a., just before free male gametes are formed, the DNA content of males is strongly increased for the formation of the eight haploid gametes (lower FACS dot plot and graph). Right panel: Mean Hoechst fluorescence intensity (DNA content) of four independent experiments. The DNA content of male Δfact-l_gam_ gametocytes at 8 m.p.a. is significantly lower than that of wild-type males (*P* = 0.0002, two-tailed two-sample *t*-test assuming unequal variance). B. Fluorescence microscopy images of Hoechst-stained male gametocytes (solid arrows) at different time points after activation, showing the increased DNA content in the enlarged male nuclei in both wild-type (wt) and Δfact-l_gam_ gametocytes compared with the haploid nuclei in ring or schizont forms (dashed arrows). In wild-type males nuclear division and gamete formation (exflagellation) are frequently observed at 12 m.p.a. whereas in Δfact-l_gam_ gametocytes the first exflagellations can be observed no earlier than 14 m.p.a. Scale bar = 6 µm. C. Percentage of exflagellating males at different time points after activation as determined by counting exflagellating males live in a Bürker-Türk counting chamber under a light microscope. Error bars represent standard deviations from three separate experiments.

After activation of gametogenesis the male gametocyte genome undergoes three successive rounds of genome duplication resulting in eight haploid genomes (Janse and Waters, 2004). These duplications are not directly followed by nuclear division and cellularization and therefore yield forms with large, octoploid nuclei within 10 min after activation. We analysed this process in activated Δfact-l_gam_ male gametocytes by FACS analyses of the DNA content of the nuclei of activated males and fluorescence microscopy of the nuclei of activated males after staining with the DNA-specific dye Hoechst-33258. Genome replication does occur in the Δfact-l_gam_ male gametocytes as shown by the strongly increased DNA content of male gametocytes at 8 and 12 min after activation visualized by FACS and fluorescence microscopy analysis (Fig. 5A). At 8 min after activation the DNA content in Δfact-l_gam_ males is lower than that of wild-type males (70%; *P* = 0.0002, using a two-tailed two sample *t*-test assuming unequal variance), while at 12 min the DNA content of the wild-type males is reduced in comparison with the Δfact-l_gam_ males, giving a value of 95% (*P* = 0.003, using a two-tailed two sample *t*-test assuming unequal variance) (Fig. 5A); although the DNA content of Δfact-l_gam_ males at 12 min is smaller than that observed for the wild-type males at 8 min (*P* = 0.1, using a two-tailed two sample *t*-test assuming unequal variance) they still contain enlarged, strongly Hoechst-positive nuclei comparable to the fully activated nuclei of male gametocytes of wild type, 8 min males (Fig. 5B).

When we analysed activated males by fluorescence microscopy we found abundant wild-type males (> 80%) in the process of forming gametes at 12 min after activation (exflagellating males with nuclear division and flagella formation; Fig. 5B), whereas such forms in Δfact-l_gam_ were only observed between 14 and 16 min after activation. When we timed exflagellation centres we observed a clear delay of approximately 3 min in the start of exflagellation between gametocytes of Δfact-l_gam_ and wild-type parasites (Fig. 5C).

Taken together, these FACS and IFA analyses show that compared with wild-type gametocytes the entire process of gamete formation is delayed in Δfact-l_gam_ males, from the onset of DNA replication through to initiation of exflagellation although Δfact-l_gam_ male gametocytes apparently do fully complete replication giving wild-type DNA content values of 8N.

Micrococcal nuclease (MNase) digestion of purified chromatin is routinely used to analyse nucleosomal DNA organization. The length of the DNA fragment associated with a histone octamer in a core particle is 148 ± 5 bp in blood-stage *P. falciparum* nucleosomes and occurs with an ≈ 160–170 bp periodicity (Lanzer *et al*., 1994). Accordingly, partially MNase-digested chromatin of schizonts and purified, unactivated gametocytes of both Δfact-l_gam_ and wild type show DNA ladders with bands consistent with the expected periodicity (Fig. S6). However, with chromatin collected from gametes 14 min after activation of enriched gametocytes, we did not observe clear nucleosome ladders even with a partial (5 min) digestion, both with wild type and with Δfact-l_gam_ parasites. Only faint mononucleosomal bands were observed with fuzzy di- and trinucleosomal ones. The absence of clear nucleosome ladders in gametes of both wild type and Δfact-l_gam_ might suggest that the DNA of *P. berghei* (male) gametes is organized in a different manner than the regular nucleosomal organization, as has also been shown for chromatin of sperm cells of other eukaryotic species (Miller *et al*., 2010). In this case the faint ‘nucleosome bands’ bands observed in the gamete samples might result from the chromatin of the female gametocytes (estimated to be ≈ 11%) or from the low contamination (≈ 10%) with chromatin of asexual blood stages, while the DNA of the male gametes randomly digested by MNase resulted in a smear.

### Δfact-l_gam_ ookinetes form oocysts that are blocked in early development

**Fig. 6:**
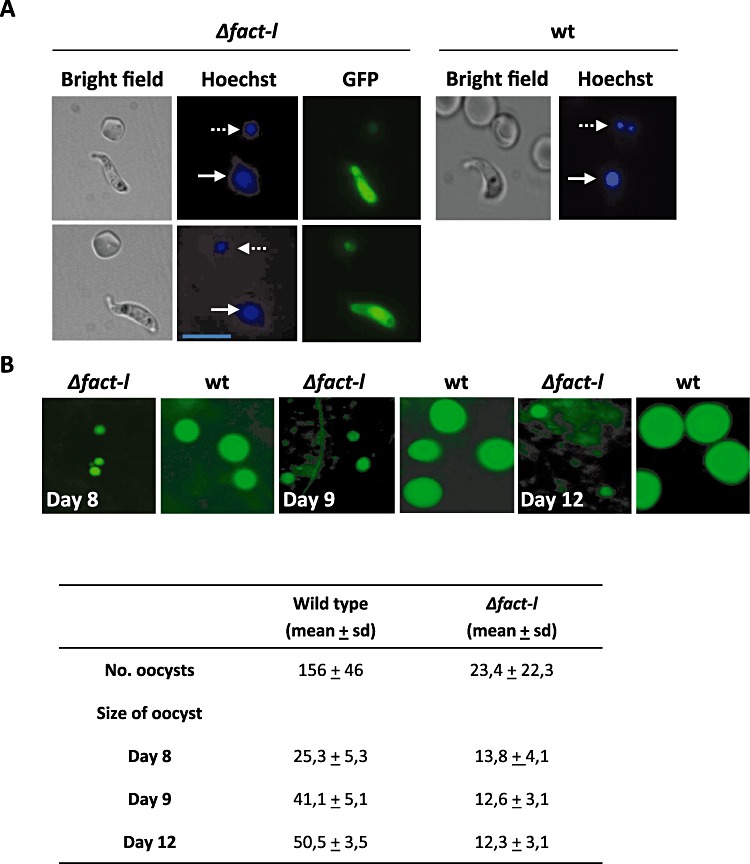
Δfact-l_gam_ parasites produce low numbers of oocysts that are blocked in sporogony. A. Although Δfact-l_gam_ produce low numbers of ookinetes (Fig. 4A), their nuclei show the typically increased fluorescence intensity after Hoechst staining (solid arrows) compared with the haploid ring forms (dashed arrows), indicating normal (DNA replication generating a 4N DNA content) meiosis comparable to wild-type ookinetes. Scale bar = 12 µm. B. Δfact-l_gam_ parasites produce low numbers of oocysts in *A. stephensi* mosquitoes that are blocked in early development as shown by the strongly reduced size. None of the oocysts shows nuclear division or sporozoite formation.

Δfact-l_gam_ parasites produce low numbers of ookinetes (see Fig. 4). Analyses of the nuclei of Hoechst-33258-stained ookinetes by fluorescence microscopy showed a strong fluorescence signal similar to that of wild-type ookinetes (Fig. 6A). This suggests a normal transition of the diploid zygote into the tetraploid ookinete as a result of DNA replication during meiosis. Mature Δfact-l_gam_ ookinetes showed normal morphology in Giemsa-stained smears but – although infective – presented clear defects in sporogony. *Anopheles stephensi* mosquitoes fed on mice infected with either Δfact-l_gam_1c or Δfact-l_gam_2c lines (four experiments with 50 mosquitoes per experiment) completely failed to generate salivary gland sporozoites (days 20–22 after feeding) nor induced blood-stage infections of naïve mice (four experiments), although low numbers of ookinetes were observed in the midguts of these mosquitoes at 18 h after feeding (results not shown). Further analysis of oocyst production and development in GFP+Δfact-l_gam_ parasites (Δfact-l_gam_2b) showed that the numbers of immature oocysts produced correlate with the reduced numbers of ookinetes (Fig. 6B). However, these oocysts remained small (Fig. 6B) and failed to produce sporozoites (days 12–20 after feeding). The small oocysts that were present at days 8–12 showed aberrant development with clustered pigment granules, no signs of nuclear division and absence of sporoblast and sporozoite formation (data not shown).

## Discussion

Transcriptome and proteome analyses of *Plasmodium* gametocytes (Florens *et al*., 2002; Lasonder *et al*., 2002; Le Roch *et al*., 2003; Hall *et al*., 2005; Khan *et al*., 2005; Silvestrini *et al*., 2005) showed that the switch from asexual to sexual development involves a significant reprogramming of transcriptional activity with a specific upregulation of as much as 25% of the ≈ 5500 *Plasmodium* genes during the formation of gametocytes, gametes and early zygote. Despite the wealth of *Plasmodium* transcriptome and proteome data (Lasonder *et al*., 2002; Le Roch *et al*., 2003; Florens *et al*., 2004; Hall *et al*., 2005; Khan *et al*., 2005; Silvestrini *et al*., 2005) the mechanisms and key players that alter gene expression during the switch from asexual to sexual development remain poorly understood. The relative scarcity of conserved transcription-associated proteins and specific *cis*-regulatory motifs recognized in the *P. falciparum* genome was thought to reflect a minor role for such mechanisms in sculpting the gene expression profile of the parasite (Gardner *et al*., 2002). However, the Apicomplexan AP2 (ApiAP2) family of putative transcriptional regulators (Balaji *et al*., 2005; De Silva *et al*., 2008) has now been clearly shown to direct stage-specific gene transcription in *P. berghei* (Yuda *et al*., 2009; 2010). In addition, there is growing evidence that epigenetic mechanisms are critical in the control and regulation of gene expression (Westenberger *et al*., 2009; Chaal *et al*., 2010; Jiang *et al*., 2010) with *Plasmodium* genomes predicted to encode a full complement of histone-modifying enzymes (Cui and Miao, 2010).

Differential accessibility to DNA for components of the transcription machinery – dynamically modulated by proteins that influence chromatin structure – plays an additional role in controlling transcription in eukaryotes as a temporary disruption of nucleosomes is required to allow progression of the transcription machinery. Such chromatin remodelling factors include the large and small FACT subunits; they also control access to DNA during replication. From the 13 bioinformatically identified chromatin remodelling proteins (Bischoff and Vaquero, 2010), nine are expressed in *P. berghei* gametocytes; five are specifically upregulated in or unique to the male gametocyte (Khan *et al*., 2005) and immunofluorescence analysis of c-Myc-tagged FACT-L in this report confirmed its abundant expression in nuclei of male gametocytes.

FACT is essential in other eukaryotes and also probably during *P. berghei* asexual blood-stage development as several attempts of standard gene disruption were unsuccessful (http://www.pberghei.eu; see RMgm 255). In the absence of technologies for efficient, conditional knock-down of such genes we developed a promoter-swap approach to specifically eliminate or substantially reduce transcription of the Spt16 large subunit of FACT (FACT-L) in sexual-stage parasites while maintaining expression in asexual blood-stage parasites to analyse the role of FACT during gameto(cyto)genesis. By Northern analysis we demonstrated a strong reduction in *fact-l*-derived mRNA in Δfact-l_gam_ gametocytes in two independent lines using two alternative promoters to control fact transcription. However, we have been unable to prove unequivocally that promoter-swap gametocytes completely lack FACT-L protein. Residual protein may be a ‘carry over’ from the early stages of gametocyte development (where they are currently indistinguishable from trophozoites) or result from leaky transcription from the chosen promoters. RT-PCR results for the mRNAs from both modified genes do not suggest *de novo* transcription implying that residual FACT-L would originate from the trophozoite stage (see also below).

### Mutant FACT females are fully fertile

The morphologically normal development of female and male gametocytes in both promoter-swap mutants indicates that fact-l transcription in the developing gametocyte is not intimately involved in the regulation of transcription of the many genes that are upregulated following the switch from asexual to sexual development (Hall *et al*., 2005; Khan *et al*., 2005). In the absence of gametocyte fact-l transcription males are still able to replicate their genomes and form axonemes (exflagellation) – albeit at a significantly slower rate – but mature gametes show reduced fertility, while female gametes are produced that are fully fertile. Hence, mutant macrogametocytes can be expected to include the majority of translationally repressed mRNAs (Mair *et al*., 2006) that accounts for 85% of the female gametocyte transcriptome (W. Hooijmaker *et al*., unpubl. data) and is essential for ookinete development. FACT plays an essential role in transcript elongation by dis- and re-assembly of chromatin and nucleosomes during transcription through interactions with histones 2A and 2B (Ransom *et al*., 2010). The lack of an effect on female gametocyte development and fertility could indicate that the nucleosome structure of chromatin in gametocyte nuclei is different from eukaryotic chromatin, although micrococcal nuclease digestion of gametocyte chromatin indicates otherwise. Therefore, a more likely possibility is that residual FACT from the initial trophozoite-like stage is sufficient for any possible chromatin modifications that are needed for transcription of gametocyte-specific genes. Alternatively, FACT plays no significant role in female gametocyte transcription.

### Mutant FACT males show delayed gametogenesis, reduced fertility and complete absence of oocyst development

Formation of fertile male gametes on the other hand depends strongly on FACT-L, probably directing correct chromatin formation during formation of the male nuclei after gametocyte activation. The rate of DNA replication is clearly impaired in Δfact-l_gam_ male gametocytes consistent with its conserved role during DNA replication in eukaryotes. In particular, human FACT cooperates with the mini-chromosome maintenance (MCM) hexameric helicase to promote the progression of replication forks (Maiorano *et al*., 2006). All known subunits of MCM are upregulated in male gametocytes including MCM4 which in humans interacts directly with FACT (Tan *et al*., 2006) (Fig. S1). The absence of this interaction in Δfact-l_gam_ males would be consistent with the reduced rate of male gametocyte DNA replication. The involvement of FACT in nucleosome disengagement from DNA during replication (Ransom *et al*., 2010) might also be slowed in its absence and contribute to the delay in DNA replication observed in our study. This delay was ultimately associated with a distinct delay in male gamete formation or exflagellation. In addition, the number of viable males (derived from ookinete conversion rates) was about six times lower in mutants than observed in wild-type parasites indicating that each male gametocyte produces an average of just over one viable male gamete. Although morphologically intact, motile and tetraploid Δfact-l_gam_ ookinetes are able to infect female *Anopheles* mosquitoes, the resulting oocysts completely fail to develop and undergo sporogony indicating a continued role for FACT-L at this stage of parasite development. This work also confirms the stage specificity of the *clag* promoter used in this study.

### Known and potential FACT-L interaction partners

FACT and NAP-1 also interact with histones H2A/H2B during DNA replication and are involved in the assembly of DNA in nucleosomes after replication (Ransom *et al*., 2010). As a result of the precedent found in *S. cerevisiae* the two FACT subunits are also expected to interact with HMG box proteins (homologues of NHP6). Surprisingly, proteome analysis of FACT-L immunoprecipitated from gametoctyes (in the absence of cross-linking and DNA shearing) revealed evidence for interactions with FACT-S only, its partner in the conserved FACT heterodimer, but neither NHP6 nor histones. However, in the same IP FACT-L co-purified with two ALBA domain-containing proteins that are enriched in male gametocytes. Together these proteins may play a role in the organization and packaging of male gamete DNA which might differ from the widely observed nucleosome-based system. In support of this, we were unable to demonstrate a clear nucleosomal pattern of DNA at 14 min after activation of purified gametocytes in wild type or promoter-swap mutants. While the vast majority of mammalian sperm chromatin is differentially sensitive to nuclease digestion (Sotolongo *et al*., 2003), spermatozoa of many higher eukaryotes replace their histones with the smaller protamines; this allows a more efficient compaction pattern of silent DNA in the male gamete nucleus (Miller *et al*., 2010; Puri *et al*., 2010) and enhances transfer to the female gamete. However, ALBA also co-purifies with P granules of *P. berghei* female gametocytes (Mair *et al*., 2006) which suggests a functional association between ALBA and RNA. Therefore the presence of two ALBA proteins in the IP could also be the result of the co-purification of proteins involved in RNA metabolism (i.e. 10 RNA-binding proteins, seven small and two large ribosomal subunit proteins) which will be established in the future.

Histones H3 and H4 are absent from the entire gametocyte proteome. Also, *Plasmodium* homologues of several factors that directly interact with histones H3/H4 in higher eukaryotes such as Asf1 (chromatin assembly protein, PFL1180w) and CAF-1 (chromosome assembly factor, PFE0090w) were either absent in the gametocyte proteomes or present at very low levels. Histones H2A/2B are more abundant in female gametocytes and asexual blood stages than in male gametocytes (Khan *et al*., 2005), indicating that the male gametocyte does not stockpile these proteins for nucleosome assembly of the eight haploid gamete genomes and that the protein extraction methods used are valid for the purification of basic proteins such as histones. Furthermore, the HMGB proteins are expressed in the nucleus in both asexual blood stages and gametocytes where they are able to interact with DNA. HMGB2 is highly transcribed in the gametocyte stage (PlasmoDB; Briquet *et al*., 2006) but predicted to be translationally repressed (Mair *et al*., 2006) as it is absent from all gametocyte proteomes (Khan *et al*., 2005), yet readily detected in the ookinete proteome (Hall *et al*., 2005). In keeping with these observations, the *Plasmodium yoelii* orthologue is redundant for asexual blood-stage development and possibly fertilization but necessary for ookinete and oocyst development (Gissot *et al*., 2008).

The expectation was that FACT and NAP function in nucleosome-based packaging of DNA synthesized during the eightfold genome replication in the male nucleus (Pace *et al*., 2006). However, the reduced fertility of Δfact-l_gam_ males (Fig. 4C), the absence of critical nucleosomal and certain chromatin assembly components, as well as evidence for the absence of a regular nucleosome packaging of gamete DNA could indicate that FACT and NAP's proteins are not involved in nucleosome formation in male gametes despite the former being central to the production of fully fertile males. Instead there is perhaps a novel role for these chromatin modifying agents in the process(es) of male gamete chromatin formation. The striking abundance of FACT and NAP in nuclei of mature male gametocytes together with the upregulation of chromatin remodelling factors SWR1 : RVB1 homologues (PF08_0100; PF13_0330) and chromatin assembly factor 1 protein WD40 domain, putative (PFA0520c), and the reduced fertility of male gametes of Δfact-l_gam_ mutants implicate these proteins in chromatin modifications that are necessary for the production of fertile gametes. Preliminary experiments in which we have disrupted the gametocyte-specific promoter of NAPs show a comparable loss of gamete fertility that were produced by male gametocytes that lack transcription of NAPs (T. Pace, I. Siden-Kiamos and C.J. Janse, unpubl. obs.).

DNA synthesis during male gametogenesis in *Plasmodium* is an extraordinarily fast process that generates ≈ 180 Mb of genomic DNA in 10 min. An understanding of the underlying key players and their molecular interactions may help identify vulnerable targets for intervention. In the light of the demonstrated and predicted essential nature of these proteins our ‘promoter-swap’ approach is a powerful tool to specifically knock-down or completely eliminate transcription in the gametocyte stage and allow the analyses of the effect of specific mutations in the domains of chromatin remodelling factors such as FACT and the NAPs. Judicious choice of other stage-specific promoters could extend this analysis to other stages of the parasite life cycle. A more general future application of promoter-swap strategies would be the analysis of the effect of stage-specific overexpression of transgenes, for example the analysis of the effect of (over)expressing mutated forms of a gene (e.g. dominant negatives). All four promoter-swap vectors reported in this study are available for use by the community.

## Experimental procedures

### *P. berghei* parasites and mice

RMgm numbers refer to mutants available in the database http://www.pberghei.eu. The following *P. berghei*, ANKA strain, reference lines were used: line cl15cy1 (Janse *et al*., 2006a) and the reference lines that stably express fluorescent reporter proteins, line 507cl1 (wt_GFP_) which expresses GFP by the constitutive eef1a promoter (Janse *et al*., 2006b; RMgm-7) and line 820cl1m1cl1 (wt-Fluo_frmg_), which expresses GFP by a male gametocyte-specific promoter and RFP by a female gametocyte-specific promoter (Ponzi *et al*., 2009; RMgm-164).

Experiments were carried out using Swiss-OF1 female mice (OF1-ico, Construct 242; age 6 weeks old; Charles River). Animal experiments in The Netherlands were performed after a positive recommendation of the Animal Experiments Committee of the LUMC (ADEC) was issued to the licensee. The Animal Experiment Committees are governed by section 18 of the Experiments on Animals Act and are registered by the Dutch Inspectorate for Health, Protection and Veterinary Public Health, which is part of the Ministry of Health, Welfare and Sport. The Dutch Experiments on Animal Act is established under European guidelines (EU directive No. 86/609/EEC regarding the Protection of Animals used for Experimental and Other Scientific Purposes).

### Generation of a transgenic *P. berghei* line that expresses a c-Myc-tagged FACT-L

For C-terminal tagging of FACT-L with a c-Myc tag, we used the standard vector pL1080 (http://www.mr4.org) as a backbone. This vector contains a double c-Myc tag. A 1.4 kb fragment of the C-terminus of fact-l was PCR-amplified using the primer pair 2779/3170 (Table S1B), subcloned into TOPO vector (Invitrogen) and subsequently transferred into SacII/BamHI-digested pL1080, resulting in vector pL1289. After linearization with XbaI this construct was transfected into *P. berghei* (ANKA) parasites of reference line cl15cy1 followed by selection and cloning of transgenic parasites (fact-l::c-myc; 1054cl1) as described (Janse *et al*., 2006a). Correct integration of the construct into the genome was analysed by diagnostic PCR, Southern blot analyses of digested genomic DNA, or chromosomes separated by field inverted gel electrophoresis (FIGE) (Janse *et al*., 2006a). FACT-l::c-Myc expression was monitored by Western analyses and immunofluorescence assay (IFA) using anti-c-Myc antibodies (see below).

### Generation of ‘promoter-swap’ mutants

To specifically eliminate or knock-down substantially fact-l transcription in gametocytes, DNA constructs were generated to exchange or ‘swap’ the endogenous fact-l promoter (Fig. 3A) with a promoter of a gene that was expected not to be transcribed in gametocytes. Vector pL0001 (http://www.mr4.org) for integration via double-cross-over homologous recombination, was used as the backbone for four different promoter-swap constructs. This vector contains the dhfr-ts gene of *Toxoplasma gondii* under the control of the *P. berghei* dhfr-ts promoter as selectable marker cassette. Three subcloning steps were performed to generate the promoter-swap constructs: first, a 539 bp fragment located 2 kb upstream of fact-l, was PCR-amplified from genomic DNA using primer pair 3190/3191 (Table S1C). This fragment represents the left flanking target region and was cloned into the KpnI/HindIII restriction sites of pL0001. As the right flanking target region, a 623 bp fragment of the open reading frame of fact-l was PCR-amplified using primer pair 3192/3193. This fragment was cloned into the SacII/XbaI restriction sites of the vector. Finally, a 2 kb fragment of the 5′ UTR promoter regions of four different genes (P1–P4) selected based on their transcription patterns in asexual blood-stage parasites was inserted. The promoter regions were amplified from genomic DNA using the primer pairs shown in Table S1C and cloned into the EcoRI/BamHI sites of the vector. The selected genes were: P1: PBANKA_091420 (PB000331.03.0; conserved *P. berghei* protein, unknown function), P2: PBANKA_140060 (PB000405.02.0; cytoadherence linked asexual protein, putative), P3: PBANKA_083690 (PB200042.00.0; BIR protein) and P4: PBANKA_011100 (PB000528.00.0; 6-cysteine protein; P12). Each promoter-swap construct (pL1312, pL1313, pL1314, pL1315) was linearized with KpnI/SacII and transfected independently into purified schizonts of *P. berghei* parasites of reference lines cl15cy1, wt_GFP_ or wt-Fluo_frmg_. Transfection, selection and cloning of ‘promoter-swap’ mutant parasites (Δfact-l_gam_ parasites) were performed as described (Janse *et al*., 2006a). Correct integration of the constructs into the genome was analysed by diagnostic PCR and Southern blot analysis of digested genomic DNA or FIGE of separated chromosomes (Janse *et al*., 2006a).

### Generation of constructs to disrupt fact-l

Attempts to disrupt fact-l are described in detail in http://www.pberghei.eu (RMgm-255) including the construct used and primers to amplify the target regions.

### Analysis of fact-L transcription and translation

Total RNA was isolated from blood-stage parasites using TRIzol reagent. To determine fact-l transcription, Northern blots of blood-stage RNA obtained from synchronized blood stages (Janse and Waters, 1995) were hybridized with a probe which was PCR-amplified with primer set 3192/3193 (Table S1C); a fact-s (PBANKA_130530) probe was PCR-amplified with primer set 3848/3913 (Table S1D). For analysis of transcription of hmgb1 (PBANKA_060190) and hmgb2 (PBANKA_071290), two specific probes were used that were PCR-amplified with primer pairs 5292/5293 and 5294/5295 respectively (Table S1D). As a loading control Northern blots were hybridized with primer L644R that hybridizes to the large subunit ribosomal RNA (van Spaendonk *et al*., 2001) or a p28-specific probe (537/538).

Epitope-tagged FACT-L (FACT-l::c-Myc) was detected with a c-Myc antibody (C3956; Sigma; dilution 1:500). As a loading control in Western analysis a rabbit polyclonal peptide antibody against a 28 kDa *P. berghei* Alba-3, PBANKA_012440 protein was used.

Nycodenz-purified gametocytes for Northern and Western analysis were obtained from infected blood of mice that had been treated with the antimalarial drug sulfadiazine to obtain highly pure populations of gametocytes (Beetsma *et al*., 1998). In these experiments mice were treated with sulfadiazine dissolved in the drinking water at a concentration of 30 mg l^−1^. Protein extracts were run on a 10% polyacrylamide gel and transferred to a nitrocellulose membrane, blocked in 3% non-fat milk powder in PBS for 1 h and probed with anti-c-MYC antibody. Primary antibody was detected by a sheep anti-mouse antibody conjugated to HRP (Horseradish Peroxidase) and (ECL-Amersham).

### Immunofluorescence analysis

For immunolocalization experiments, air-dried smears were fixed with ice-cold methanol (Wickham *et al*., 2001). Anti-c-Myc antibody was diluted in PBS containing 3% BSA (C3956; Sigma; dilution 1:400). Parasite nuclei were stained with 1.5 mg ml^−1^ DAPI for 2 min (Vector Laboratories). Images were taken using a Leica DMR microscope and a 100×/PL FLUOTAR objective, and pictures recorded using a CoolSNAP HQ^2^ (Photometrics, the Netherlands) digital camera. Images were processed using Adobe Photoshop.

### IP experiments and mass spectrometric analysis

Immunoprecipitations of FACT-L::c-Myc were performed essentially as described (Mair *et al*., 2006) using whole-cell lysates from purified gametocytes and asexual blood-stage parasites. Infected red blood cells were washed three times with ice-cold ‘enriched’ PBS, lysed in 250 µl of NET-2 [50 mM NaCL, 150 mM TrisHCl (pH 7.4), 0.5% NP40] containing 2 mM DTT and supplemented with protease inhibitors (Complete Mini; Roche) and RNasin (2 units µl^−1^; Promega). After lysis the extract was spun for 10 min at 14 000 r.p.m. to remove the pelleted, insoluble fraction. Extract of 50 µl was used per IP with anti-GFP antibody (1 µg, Roche), anti-c-myc antibody (2.5 µl, Sigma) or beads-only and incubated on ice for 1 h with occasional mixing. Recovery of proteins was performed with protein G-sepharose beads (20 µl packed bead volume per IP) in a total volume of 200 µl for 1 h on ice with occasional mixing. IPs were washed four times with 400 µl of NET-2 and divided in two equal volumes. One pellet was resuspended in SDS-PAGE loading buffer and used for Western analysis or for mass spectrometry. Mass-spectrometric analysis was performed by LTQ-FT was performed as described (Mair *et al*., 2010).

### *In vitro* (cross-)fertilization/ookinete maturation assays

Gametocyte production was determined in synchronized *in vivo* infections as described (Janse and Waters, 1995). The gametocyte conversion rate is the percentage of ring forms developing into gametocytes under standardized conditions. The fertility of gametes was analysed by standard *in vitro* fertilization and ookinete maturation assays (van Dijk *et al*., 2010). Gametocytes for these assays were obtained either from infected mice that had been pre-treated with phenylhydrazine to increase gametocyte numbers, or from infected mice that had been treated with the antimalarial drug sulfadiazine (dissolved in the drinking water at a concentration of 30 mg l^−1^) to obtain highly pure gametocyte populations (Beetsma *et al*., 1998). The ookinete conversion rate is defined by the percentage of female gametes that develop into mature ookinetes under standardized *in vitro* culture conditions for activation of gametocytes, fertilization and ookinete maturation. The percentage of females that developed into ookinetes was determined by counting female gametes and mature ookinetes in Giemsa-stained blood smears, made at 16–18 h post activation (hpa) of gametocytes or by counting the ratio of GFP-positive, unfertilized female gametes and GFP-positive ookinetes at 16–18 hpa by fluorescence microscopy in line Δfact-l_gam_ 1b and Δfact-l_gam_ 2b.

Fertility of individual sexes (male and female gametes) of the different mutant lines was determined by *in vitro* cross-fertilization studies in which gametes are cross-fertilized with gametes of lines that produce only fertile male (Δp47; 270cl1; RMgm-348) or only fertile female gametes (Δp48/45; 137cl8; RMgm-15) (van Dijk *et al*., 2010). Fertilization and ookinete maturation assays were always performed in triplicate, and on multiple occasions in independent experiments.

### Analysis of fertilization and meiosis by FACS

Fertilization and meiosis in all lines was analysed using standard *in vitro* fertilization and ookinete maturation assays (van Dijk *et al*., 2010). Fertilization and meiosis were inferred from DNA content (or ploidy) by FACS measurement of the fluorescence intensity of female gametes and zygotes stained with the DNA-specific dye Hoechst-33258 (Janse *et al*., 1987; Janse and Van Vianen, 1994). For these experiments we used the mutant lines Δfact-l_gam_ 1c (1134cl1) and Δfact-l_gam_ 1c (1135cl1) and Δp48/45-fluo (1197cl1; RMgm-345) which are all generated in the parent line wt-Fluo_frmg_ (820cl1m1cl; RMgm-164) that expresses RFP in the female gametocyte/gamete and during further development into the zygote/ookinete (Ponzi *et al*., 2009; Mair *et al*., 2010). The stage-specific RFP expression allows selection of female gametes and zygotes stages in the process of FACS analysis of the DNA content of cells (see Fig. 4B). Activation of gametocytes was performed in *in vitro* (cross-)fertilization/ookinete maturation assays as described above. At 4 hpa cells were stained for 1 h at room temperature with Hoechst-33258 (10 mM) and subsequently analysed by FACS using a LSR-II flow cytometer (Becton Dickinston). Cells were analysed at room temperatures with the following filters (parameters/thresholds): UB 440/40 (Hoechst) (400/5000); BE 575/26 (RFP) (500/5000); BF 530/30 (GFP) (500/5000); FSC (250/2000); SSC (200/5000). The cells for analysis were selected on size by gating on FSC and SSC. A total of 10 000–500 000 cells were analysed per sample and all measurements were performed on triplicate cultures. Subsequently the unfertilized female gametes (1N) and zygotes (4N) were selected based on their RFP expression. To determine the Hoechst-fluorescence intensity from the populations of unfertilized female gametes and zygotes, gates were set as shown in the Fig. 4B. The fertilization rate is defined as the percentage of female gametes that developed into zygotes. Data processing and analysis was performed using the program FlowJo (http://www.flowjo.com).

### Micrococcal nuclease digestion of nuclei of schizonts and purified gametocytes

Purified schizonts (0.5–15 × 10^9^) were obtained as described (Janse *et al*., 2006a). Purified mature gametocytes (0.5 × 10^9^) and activated gametocytes (0.5 × 10^9^) were prepared as described above. Activated gametocytes were collected at 14 min after resuspending in gametocyte/ookinete activation medium. Purified schizonts and gametocytes were resuspended in 3 ml of cell lysis buffer (10 mM Tris pH 8; 3 mM MgCl_2_; 0.2% Nonidet P-40; protease inhibitors Complete Mini-Roche) and gently homogenized with a dounce homogenizer (Kontes, pestle B) on ice (≈ 30 strokes). After lysis, nuclei were separated by centrifuging (2000 r.p.m., for 10 min at 4°C) through a 0.25 M sucrose cushion and subsequently washed in 3 ml of digestion buffer (50 mM Tris pH 7.4; 4 mM MgCl_2_; 1 mM CaCl_2_; protease inhibitors Complete Mini-Roche), centrifuged at 2000 r.p.m. for 10 min at 4°C and resuspended in 150 µl of digestion buffer. The digestion was carried out by adding 1 µl of micrococcal nuclease (0.5 U µl^−1^) for 8 or 5 min at 37°C and was stopped by adding 10 mM EDTA (final concentration). DNA was isolated by column purification (Qiagen, PCR purification kit) and analysed on 1.5% agarose gels.

### Analysis of DNA synthesis/content of activated male gametocytes and exflagellation

DNA synthesis/content in male gametocytes after activation was analysed using standard *in vitro* fertilization and ookinete maturation assays (van Dijk *et al*., 2010). Purified gametocytes (see above) were transferred to standard ookinete culture medium (RPMI1640; pH 8.0; 21°C; Janse *et al*., 1985) in Eppendorf tubes for activation of gamete formation. At 0, 2, 8 and 12 min after activation cells were pelleted by centrifugation (5 s at 10.000 r.p.m.; Eppendorf centrifuge), fixed in 0.25% glutaraldehyde/PBS solution and stained with 2 µM Hoechst-33258 (Janse *et al*., 1987). The Hoechst-fluorescence intensity (DNA content) of the gametocytes were analysed by FACS using a LSR-II flow cytometer (Becton Dickinston). Cells were analysed at room temperature with the following filters (parameters/thresholds): UB 440/40 (Hoechst) (400/5000); FSC (250/2000); SSC (200/5000). The cells for analysis were selected on size by gating on FSC and SSC. A total of 10 000–500 000 cells were analysed per sample and all measurements were performed on triplicate samples. To determine the Hoechst-fluorescence intensity (DNA content) from the populations of activated female and male gametocytes, gates were set as shown in Fig. 5A. Data processing and analysis was performed using the program FlowJo (http://www.flowjo.com). Exflagellation was determined in the same cultures as described above at different time points (10–22 min) after activation of gamete formation. Exflagellations were counted in Bürker-Türk counting chamber under a light microscope. Exflagellations were counted in parallel in wild-type and promoter-swap mutant parasites, activated at the same time, in the same culture medium at the same temperature (21°C). Hoechst-33258-stained activated male gametocytes were analysed in methanol fixed smears using a Leica DMR microscope and a 100×/PL FLUOTAR objective, and pictures recorded using a CoolSNAP HQ^2^ (Photometrics, the Netherlands) digital camera. Images were processed using Adobe Photoshop.

### Mosquito transmission experiments

For mosquito transmission experiments female *Anopheles stephensi* mosquitoes were fed on mice infected with wild-type parasites or promoter-swap mutants. Oocyst development, oocyst production and sporozoite production was monitored in infected mosquitoes as described (Sinden, 1997). Oocyst and sporozoites numbers were counted in infected mosquitoes at 6–12 days and 21–22 days after infection. The size of oocysts was measured from wild-type and mutant parasites in parallel using pictures of midguts containing GFP-positive oocysts recorded using a CoolSNAP HQ^2^ (Photometrics, the Netherlands) digital camera on a Leica DMR microscope.

## Supporting information

**Fig. S1.** Immunoprecipitation of FACT-L::c-Myc with anti-c-Myc antibodies and mass-spectrometric (LC-MS/MS) analysis of co-precipitated proteins.

A. Western blot analyses of IPs of lysates from purified gametocytes with anti-c-Myc, anti-GFP or beads only, showing the presence of FACT::c-myc only in the specific fraction.

B. Proteins identified by mass-spectrometric (LC-MS/MS) analysis of proteins co-precipitated with FACT-L::c-myc after immunopurification with a monoclonal anti-c-Myc antibody. Those proteins detected in the FACT-L IP are shaded in orange and represent values obtained by IP with either anti-GFP (control) or anti-C-Myc monoclonal antibodies. No peptides were detected for HMGBP1 and 2 in this analysis (unshaded). The two right columns indicates the cumulative peptide counts for HMGBP and for the annotated members of the MCM complex from a previously published LC-MS/MS analysis of purified male (blue shading) and female (pink shading) gametocytes (Khan *et al*., 2005).

**Fig. S2.** CLUSTALW alignment of Spt16 subunit of the FACT complex. The three conserved domains (peptidase_M24; Spt16, Rtt106) are indicated by the green bars.

**Fig. S3.** CLUSTALW alignment of SSRP1 subunit of the FACT complex. The conserved domain (structure-specific recognition protein signature) is indicated by the green bar.

**Fig. S4.** CLUSTALW alignment of NHP6 of *S. cerevisiae* with the HMGB proteins of *Plasmodium berghei* and the C-terminus of SSRP1 of *H. sapiens*. The conserved class II high-mobility group (HMG) protein box is underlined inred.

**Fig. S5.** Immunofluorescence of wild-type parasites stained with anti-c-Myc antibody. A. The subcellular localization of Fact-L is shown by immunofluorescence analysis of blood stages of fact-l::c-myc parasites using anti-c-Myc antibodies. FACT-L (green) colocalizes with the DAPI-stained nuclei (blue) of rings, trophozoites (T) and schizonts. B. The backgroundcytoplasmic staining c-Myc (green) is shown by immunofluorescence analysis of blood stages of wild-type parasites using anti-c-Myc antibodies. It is greatly reduced compared with the mutant parasitelines (Figs 2C and S5A); however, the apical end of segmented merozoites in schizont stages does react with the antibody. DAPI-stained nuclei (blue) of rings, schizonts and gametocytes. Scale bars = 6 µm.

**Fig. S6.** Micrococcal nuclease digestion of nuclei of schizonts (sch) and purified gametocytes (gam) of wild-type (wt) and Δfact-lgam parasites. Left panel: schizonts and gametocytes pre-activation. Right panel: gametocytes 12 min post activation (digestion time 5 or 8 min).

**Table S1.** Sequence of oligonucleotide primers used in this study.
