# Supplementary figures and images for: Experimentally controlled downregulation of the histone chaperone FACT in Plasmodium berghei reveals that it is critical to male gamete fertility
Source: Cell Microbiol. 2011 Dec;13(12):1956–74. doi: 10.1111/j.1462-5822.2011.01683.x (PMC3429858; doi:10.1111/j.1462-5822.2011.01683.x)

## Slide 1
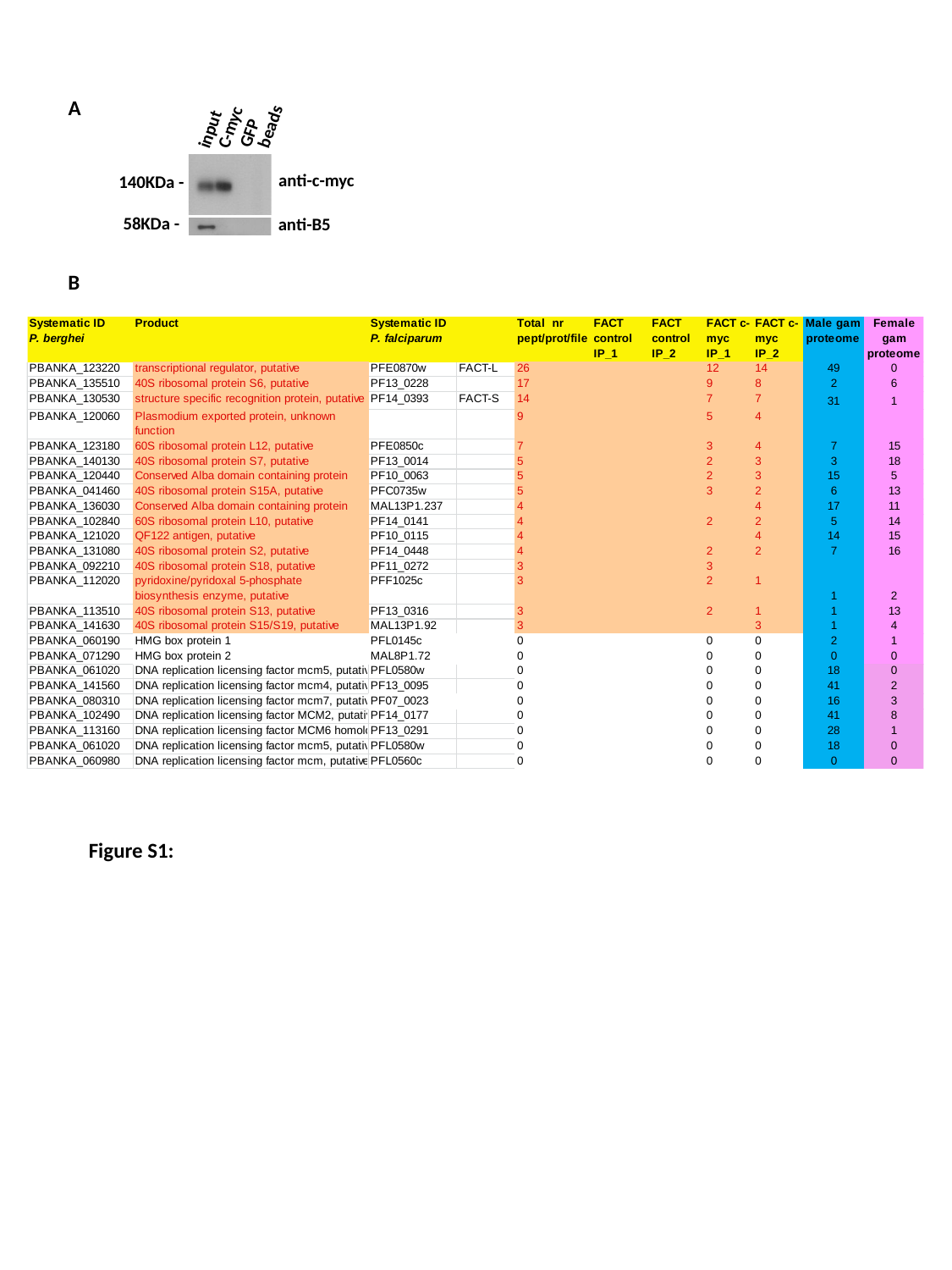

A
beads
C-myc
GFP
input
anti-c-myc
140KDa -
58KDa -
anti-B5
B
Figure S1:

Supplement: Supplementary file 1 [file cmi0013-1956-SD2.pptx]

## Slide 1
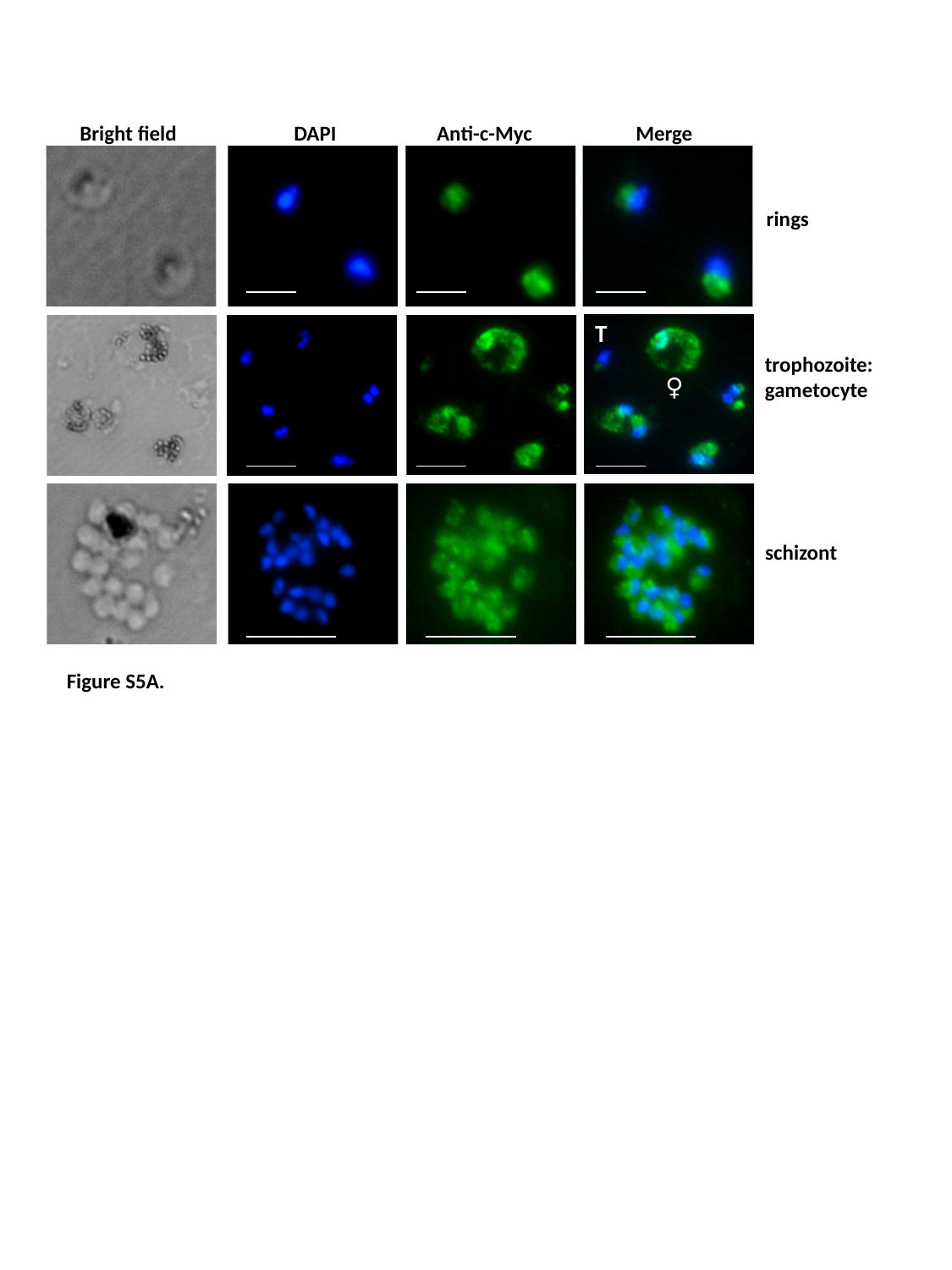

Bright field
DAPI
Anti-c-Myc
Merge
rings
T
trophozoite: gametocyte
♀
schizont
Figure S5A.

Supplement: Supplementary file 5 [file cmi0013-1956-SD6.pptx]

## Slide 1
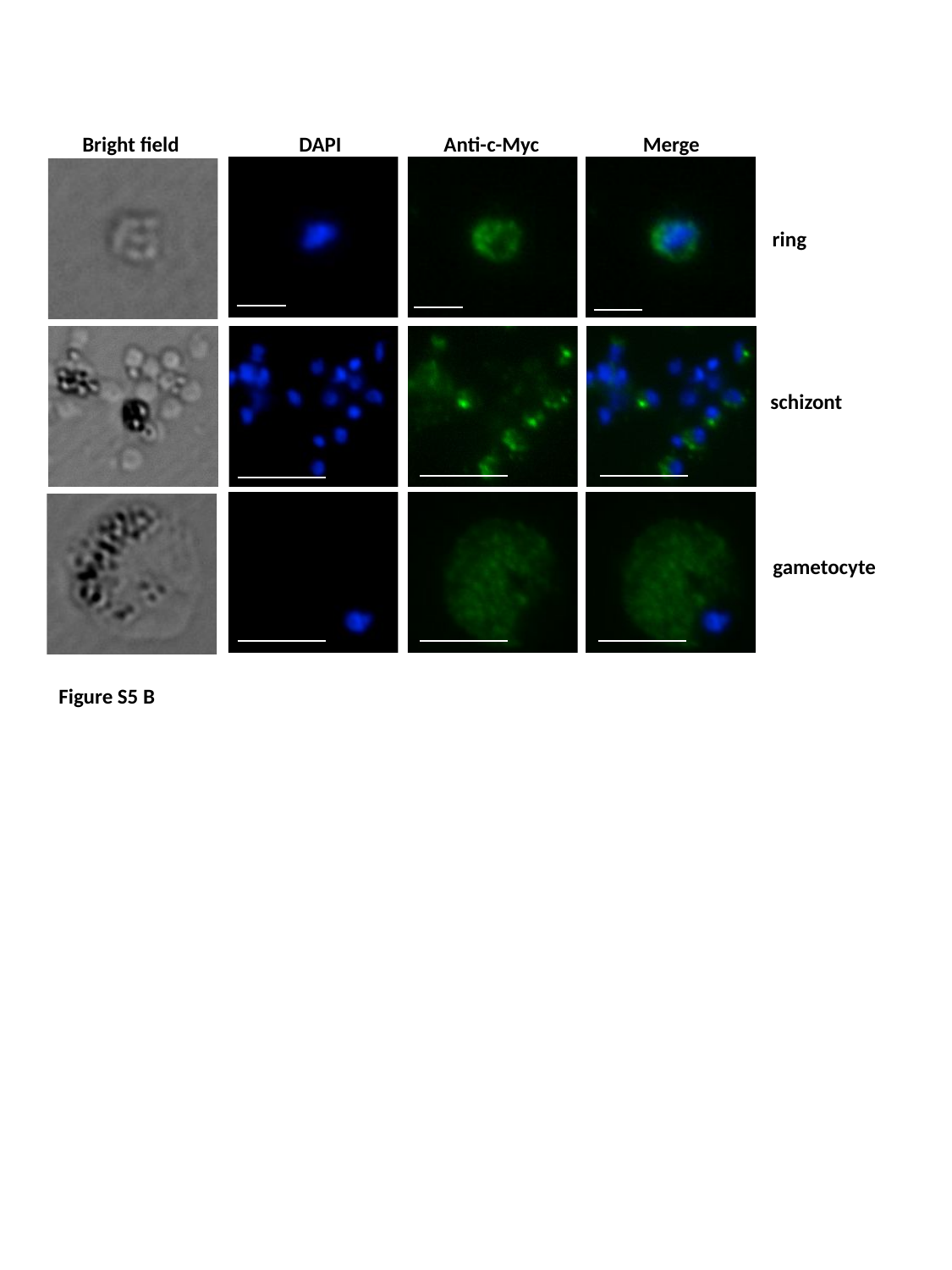

Bright field
DAPI
Anti-c-Myc
Merge
ring
schizont
 gametocyte
Figure S5 B

Supplement: Supplementary file 6 [file cmi0013-1956-SD7.pptx]

## Slide 1
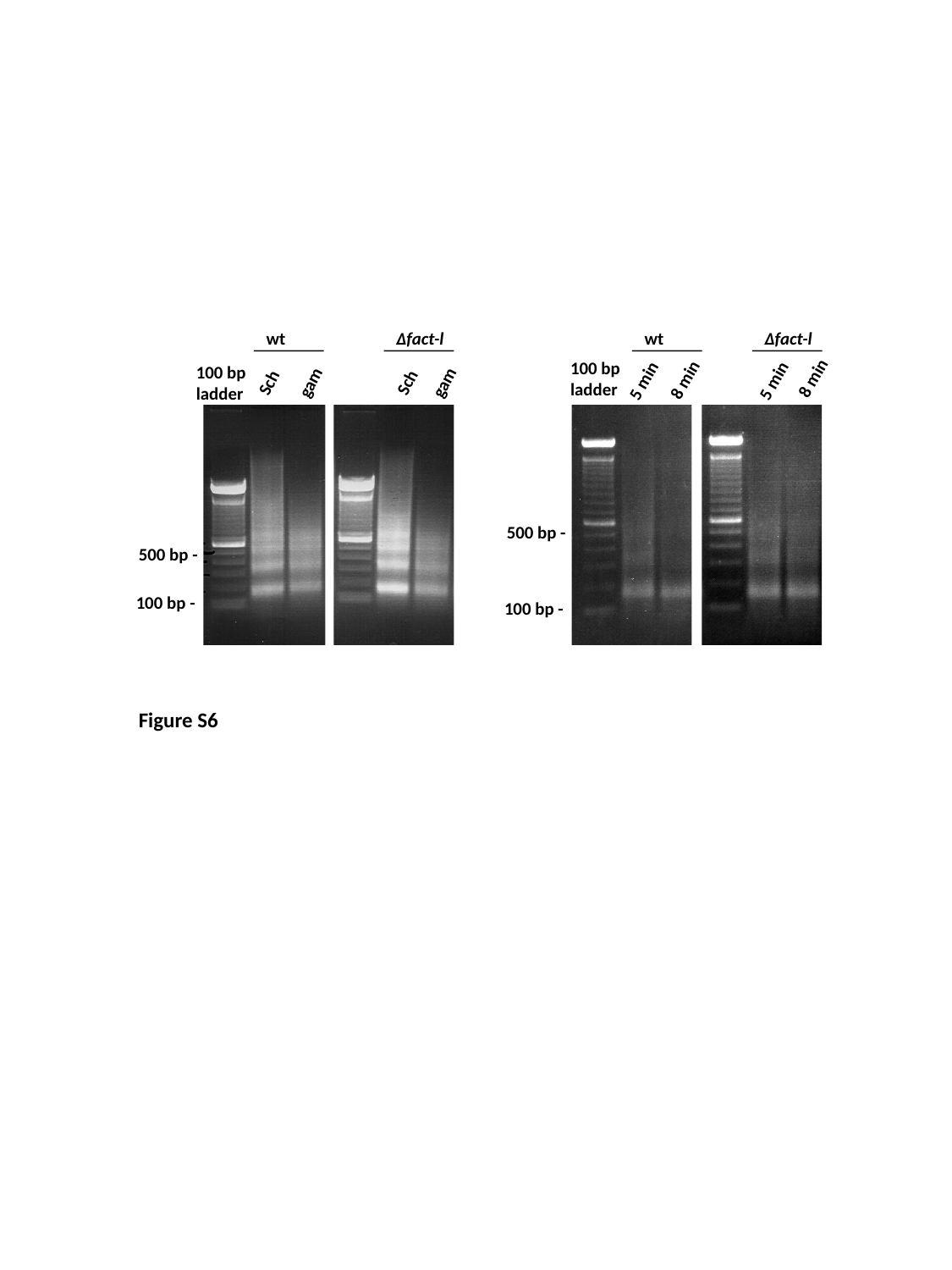

wt
Δfact-l
wt
Δfact-l
8 min
8 min
100 bp ladder
gam
gam
100 bp ladder
5 min
5 min
Sch
Sch
500 bp -
500 bp -
100 bp -
100 bp -
Figure S6

Supplement: Supplementary file 7 [file cmi0013-1956-SD8.ppt]
