# Supplementary material for: Experimentally controlled downregulation of the histone chaperone FACT in Plasmodium berghei reveals that it is critical to male gamete fertility
Source: Cell Microbiol. 2011 Dec;13(12):1956–74. doi: 10.1111/j.1462-5822.2011.01683.x (PMC3429858; doi:10.1111/j.1462-5822.2011.01683.x)
Supplement: Supplementary file 2 [file cmi0013-1956-SD3.rtf]

Figure S2
 


PBANKA_123220_prot	1-MSEGLDVDNAKEKINFMFTYWKNINNKDFENSNAFCILSGKSSKDDNATIQEQFQMWLL
PFE0870w_prot         1 -MSEALDIDNAKAKIGLVFSYWKKVANNDFSKCSVFCVLSGKSSKDENATIQEQFQMWLT
YGL207W_prot          1 MEELNIDFDVFKKRIELLYSKYNEF--EGSPNSLLFVLGSS--NAENPYQKTTILHNWLL
gi_6005757_hFACT      1 -MAVTLDKDAYYRRVKRLYSNWRKG--EDEYANVDAIVVSVGVDEEIVYAKSTALQTWLF
consensus             1  .....*.*...... ......... .... ........*........ . . . ..**.


PBANKA_123220_prot   60 GYQLTETFFLFCKKNEKLIILTSDKKKKFLQPLLDKMNN---------ITILERNNNDNS
PFE0870w_prot        60 GYQLTETFFVFLKNSERILILTSDKKKRFLQPLLDNIKN---------VDVLERSN-DNT
YGL207W_prot         57 SYEFPATLIALVPG--KVIIITSSAKAKHLQKAIDLFKDPESKITLELWQRNNKEPELNK
gi_6005757_hFACT     58 GYELTDTIMVFCDD--KIIFMASKKKVEFLKQIANTKGNENANGAPAITLLIREKNESNK
consensus            61 .* ...*......  .......*..*...*..... ...        .. .... ...*.


PBANKA_123220_prot  111 ENFEKIKNEINMFNDKELLILKDKDSTGSFFEACY-----DFIKNLNKTEIDVNNNIKSL
PFE0870w_prot       110 SNFENIKSTIESTNCDEIALLKDKDATGSFFENCY-----DFIKTLNKKEMDVNNNIKEL
YGL207W_prot        115 KLFDDVIALIN--SAGKTVGIPEKDSYQGKFMTEWNPVWEAAVKENEFNVIDISLGLSKV
gi_6005757_hFACT    116 SSFDKMIEAIKESKNGKKIGVFSKDKFPGEFMKSWN----DCLNKEGFDKIDISAVVAYT
consensus           121 ..*...   *. .. . ......**... .*  ...    .... ..  ..*. .... .


PBANKA_123220_prot  166 LNLRSKSDVKLQKSASDIASIIMKNVLITTIENSLDSEEYESHNKIKEKVLKFNENKKCV
PFE0870w_prot       165 LNFRSDTDMKIQKSGSDIACIILKSILITTIENALDNEEFESHDKIKEKALKFMDNKKCV
YGL207W_prot        173 WEVKDVNEQAFLSVSS-KGSDKFMDLLSNEMVRAVDEELKITNAKLSDKIENKIDDVKFL
gi_6005757_hFACT    172 IAVKEDGELNLMKKAASITSEVFNKFFKERVMEIVDADEKVRHSKLAESVEKAIEEKKYL
consensus           181 ........................ ..........* ...... *..... ......*..


PBANKA_123220_prot  226 VKIKDKLKADIDDIDVIYSS---------VQSGNNFLLNFKNTNDNNYLSQNDGTIVIGI
PFE0870w_prot       225 MKLKDKLKVDIEEIDVIYSN---------VQSGNNFTLTYKNSNDKNYLSQNEGTILVGV
YGL207W_prot        232 KQLSPDLSALCPPNYKFNFDLLDWTYSPIIQSGKKFDLRVSARSTNDQLYG-NGCILASC
gi_6005757_hFACT    232 AGADPSTVEMCYP--------------PIIQSGGNYNLKFSVVSDKNHMH--FGAITCAM
consensus           241 .... ..... ........        ...***... *.. .. . .......*.*....


PBANKA_123220_prot  277 GVKYKELCANINRTLLLNAKEYHKELYNFTFSIQKYIINDCLKYNTSFSDVYKKAMQYIK
PFE0870w_prot       276 GLKYKELCCNITRTLLLNARTQHKELYNFTISIEKYIIKECLKVGTNFSSVYKKTLEYVK
YGL207W_prot        291 GIRYNNYCSNITRTFLIDPSEEMANNYDFLLTLQKEIVTNILKPGRTPKEVYESVIEYIE
gi_6005757_hFACT    276 GIRFKSYCSNLVRTLMVDPSQEVQENYNFLLQLQEELLK-ELRHGVKICDVYNAVMDVVK
consensus           301 *..... *.*..**...  ...... *.* ..... .....*. ......**........


PBANKA_123220_prot  337 DNKRNYQTIGNINLENYFIKCLGHVIGFEFMEKEFLITVNNSNATIEKNTSYNISVGFEN
PFE0870w_prot       336 EHKKEYKTLSNIQIENYFVKCIGHIIGIEFIDKEYLIIESNHQGKIQKNTSYNLSVGFEN
YGL207W_prot        351 KTKP--------ELVPNFTKNIGSLIGLEFRDSNFILNVKNDYRKIQRGDCFNISFGFNN
gi_6005757_hFACT    335 KQKP--------ELLNKITKNLGFGMGIEFREGSLVINSKNQY-KLKKGMVFSINLGFSD
consensus           361 ..*. . .. .........* .*...*.**..........* ...... .......**..


PBANKA_123220_prot  397 VQMPDSKN----VFSTWISDTVFVN-DKDEITILTDAISKEINTISYELEDSGSENEE--
PFE0870w_prot       396 VQGLE-KN----KFAIWISDTICID-DNEDVIVLTDAISKEINTISYELEDTKSDDEEGD
YGL207W_prot        403 LKDSQSAN----NYALQLADTVQIPLDETEPPRFLTNYTKAKSQISFYFNNEEEDNNK--
gi_6005757_hFACT    386 LTNKEGKKPEEKTYALFIGDTVLV--DEDGPATVLTSVKKKVKNVGIFLKNEDEEEEE--
consensus           421 ..  ....     ......**. .  *.... ..  ...*.......... .. ....  


PBANKA_123220_prot  450 ----------------------ENSEDNKKKNIKESKNVKREG-----------------
PFE0870w_prot       450 DDERDDDDEKDDERDDERDDEKDDDEDDEKKKKKKKKNVKSEKGVSVKKEKKNKHNNSHK
YGL207W_prot        457 ------------------------------------------------------------
gi_6005757_hFACT    442 ------------------------------------------------------------
consensus           481                       .  ..  ..  .  .... .                  


PBANKA_123220_prot  471 -------SDYNESDDDDRNKKSEKVKKEKKKNEDNENKKKIGISASILNNASSVIVSDRL
PFE0870w_prot       510 YDDNDDEEDDDEDDDDDNDDDDDDNNNNNNNNGLHKDKKKTGISASILNNAASVIVSDRL
YGL207W_prot        457 --------------------------------------KKSSPATKVPSKPDRNSKILRT
gi_6005757_hFACT    442 --------------------------------------EEKDEAEDLLGRGSRAALLTER
consensus           541         .  . ....     .        .     ...... ........ .......

PBANKA_123220_prot  524 RRRNKNSLAHNNEQEIEELNKRQNELKNKKIEEIKNRFSEGTNEYKDLNKKNIKKLEDIK
PFE0870w_prot       570 RRRNKNSLAHNNEQEMEELNKRQHELKEKKINDIKIRFSKGTNDYKDLNKKNIKKLEDLK
YGL207W_prot        479 KLRGEARGGAEDAQKEQIRKENQKKLHEKLEKNGLLRFSAADANGPDSEPR--QYFKKYE
gi_6005757_hFACT    464 TRN--------EMTAEEKRRAHQKELAAQLNEEAKRRLTEQKGEQQIQKAR--KSNVSYK
consensus           601 .................. ...*..*... ......*.......................


PBANKA_123220_prot  584 SYNDADLLPRDLRSNIIHVDNKHESILLPVNGAHIPFHVSTIKNLSSNYEDNNDIFVLRI
PFE0870w_prot       630 TYNDPDLLPKDLRPNIICVDNKHECILLPINGLHIPFHVSTIKNLSSNYEDNNDIFVLRI
YGL207W_prot        537 SYVRDSQLPTNIRDLRIHVDWKSQTIILPIYGRPVPFHINSYKNGSKNEE--GEYTYLRL
gi_6005757_hFACT    514 NPSLMPKEP-HIREMKIYIDKKYETVIMPVFGIATPFHIATIKNISMSVE--GDYTYLRI
consensus           661 .... ...*...*...*..*.*......*..*...***....**.*...*.. .   **.


PBANKA_123220_prot  644 NFQVPGNQG-------SQKGEFNSFPKLNEKEMYIKELIFKSSDEKHLQILVKQVKELIK
PFE0870w_prot       690 NFLVPGNQG-------VVKGELNTFPTLQQNQMYIRELIFKSPNEKHFQMVVKQVKELIK
YGL207W_prot        595 NFNSPGSSGG------ISKKVEELPYEESADNQFVRSITLRSKDGDRMSETFKQIADLKK
gi_6005757_hFACT    571 NFYCPGSALGRNEGNIFPNPEATFVKEITYRASNIKAPGEQTVPALNLQNAFRIIKEVQK
consensus           721 **..** ...      . ... ......  ............ ........ .......*


PBANKA_123220_prot  697 QVKQKEVEADVNDSKTSNEKLALNKTGRRIVLRDLMTRPNIFTGRKILGTLELHTNGLRY
PFE0870w_prot       743 QVKQKEVEADVNESKTSQDRLVLNKSGRRIVLRDLMTRPNIFTGRKILGTLELHMNGLRY
YGL207W_prot        649 EATKREQERKALADVVQQDKLIENKTGRTKRLDQIFVRPNPDT-KRVPSTVFIHENGIRF
gi_6005757_hFACT    631 RYKTREAEEKEKEGIVKQDSLVINLNRSNPKLKDLYIRPNIAQ-KRMQGSLEAHVNGFRF
consensus           781 .....*.*. ..... ....*..*.......*.....***.............*.**.*.


PBANKA_123220_prot  757 SANSRGTTEYIDILFDDIKHAFYQPCDGQLIILIHFHLKRYIMVGKKKTLDVQFYCEVGT
PFE0870w_prot       803 AANSRGTTEFIDILFDDIKHAFYQPCDGQLIILIHFHLKRYIMVGKKKTLDVQFYCEAGT
YGL207W_prot        708 QSP-LRTDSRIDILFSNIKNLIFQSCKGELIVVIHIHLKNPILMGKKKIQDVQFYREASD
gi_6005757_hFACT    690 TS---VRGDKVDILYNNIKHALFQPCDGEMIIVLHFHLKNAIMFGKKRHTDVQFYTEVGE
consensus           841 . ....... .***.. **....*.*.* .*...*.*** .*..***...*****.* ..


PBANKA_123220_prot  817 QIDDLDR------AKARNVYDPDEMHDEMKEREQKNKLNLIFKNFVQQMQDIS--KIEFE
PFE0870w_prot       863 QIDDLDR------AKARNVYDPDEMHDEMKEREQKNKLNLIFKNFVQQMQDIS--KIEFE
YGL207W_prot        767 MSVDETGGGRRGQSRFRRYGDEDELEQEQEERRKRAALDKEFKYFADAIAEASNGLLTVE
gi_6005757_hFACT    747 ITTDLG--------KHQHMHDRDDLYAEQMEREMRHKLKTAFKNFIEKVEALTKEELEFE
consensus           901 ...*...      .......*.*....* .**.....*...**.*........  ....*


PBANKA_123220_prot  869 IPYPELTFSGVPNKSNVEIFVTANTINHLIEWPPFILSVEDIEIASLERVHHGLRNFDMI
PFE0870w_prot       915 IPYPELTFSGVPNKSNVEIFVTANTINHLVEWPPFILSVEDIEIASLERVHHGLRNFDMI
YGL207W_prot        827 NTFRDLGFQGVPNRSAVFCMPTTDCLVQLIEPPFLVINLEEVEICILERVQFGLKNFDMV
gi_6005757_hFACT    799 VPFRDLGFNGAPYRSTCLLQPTSSALVNATEWPPFVVTLDEVELIHFERVQFHLKNFDMV
consensus           961 ... .* *.*.*..*..... *.... ...*.*.........*....***  .*.****.


PBANKA_123220_prot  929 FVFKDYTKPVKRIDVIPVEYIDTIKKWLTTIDIVYYEGKNNLQWGNILKTILADIESFVN
PFE0870w_prot       975 FVFKDYTKPVKRIDVIPTEYIDTIKKWLTTIDIVYYEGKNNLQWGNILKTILSDIDSFVN
YGL207W_prot        887 FVYKDFNKPVTHINTVPIESLDFLKQWLTDMDIPYTVSTINLNWATIMKSLQDDPYQFFL
gi_6005757_hFACT    859 IVYKDYSKKVTMINAIPVASLDPIKEWLNSCDLKYTEGVQSLNWTKIMKTIVDDPEGFFE
consensus          1021 .*.**..*.* .* ..*.. .*..*.**...*..* .....*.*..*.*....* ..* .


PBANKA_123220_prot  989 SKGFDGFLGEDDDEEEQSAEDEDEDDEYEVDESEMSAEEDSEYEDSEEESLATE---SDG
PFE0870w_prot      1035 SKGFDGFLGEDDDEEEETADDEDEDDEYEVDESELSAEEDSEYDDSEDESLATE---SDG
YGL207W_prot        947 DGGWNFLATGSD----DEASDESEEEVSEYEASEDDVSDESAFSEDEEGSEVDDDISGDE
gi_6005757_hFACT    919 QGGWSFLEPEGEGSDAEEGDSESEIEDETFNPSEDDYEEEEEDSDEDYSSEAEE---SDY
consensus          1081 . *..  ..............* *........**. .............* ...   .*.
                                       DELEERAKKDDKKRFAYQSDDGDDSEGYNKRKRKKN

PBANKA_123220_prot 1046 DEEVEEDSDDEGLSWDELEERAKKGKPQYIQ-----------------------------
PFE0870w_prot      1092 DEEVEEDSEDEGLSWDELEERAKKDDKKRFAYKSDEDDEGYNKRKKKKKN----------
YGL207W_prot       1003 SEDYTGDESEEGEDWDELEKKAARADRGANFRD---------------------------
gi_6005757_hFACT    976 SKESLGSEEESGKDWDELEEEARKADRESRYEEEEEQSRSMSRKRKASVHSSGRGSNRGS
consensus          1141  .... . ...*. *****..*.....   . . ..      ....              

PBANKA_123220_prot      ------------
PFE0870w_prot           ------------
YGL207W_prot            ------------
gi_6005757_hFACT   1036 RHSSAPPKKKRK
consensus          1201             
