# Supplementary material for: Experimentally controlled downregulation of the histone chaperone FACT in Plasmodium berghei reveals that it is critical to male gamete fertility
Source: Cell Microbiol. 2011 Dec;13(12):1956–74. doi: 10.1111/j.1462-5822.2011.01683.x (PMC3429858; doi:10.1111/j.1462-5822.2011.01683.x)
Supplement: Supplementary file 3 [file cmi0013-1956-SD4.rtf]

Figure S3


PBANKA_130530_prot    1 MPADNTGSTSSNSPVISIGNIRGLGGCDYGPFRMSNEFLGWKNKKTN--------SVYQY
PF14_0393_prot        1 --MGDNNTSSGSSPVLSVGNIRGFGGSDFGSFRMSNEFLGWKNKKTN--------NVYQY
YML069W_prot          1 -------------MSTDFDRIYLNQSKFSGRFRIADSGLGWKISTSGGSAANQARKPFLL
Q08945_SSRP1_HUMAN    1 ---------MAETLEFNDVYQEVKGSMNDGRLRLSRQGIIFKNSKTG--------KVDNI
consensus             1        .... ........... .  ..*..*.... ...*. ..         .....


PBANKA_130530_prot   53 KCNDISEGEWIKLSYNNNRLHLKFNESKDNLIVFFDGFPDRNLSEITQHFQKYFNIKLGT
PF14_0393_prot       51 KCSDIDEGCWIKTSYNNNRLHLKLGESKENIIIYFDGFPDRNVNEITQHFQKYFNIRLNN
YML069W_prot         48 PATELSTVQWSRGCRG----YDLKINTKNQGVIQLDGFSQDDYNLIKNDFHRRFNIQVEQ
Q08945_SSRP1_HUMAN   44 QAGELTEGIWRRVALG----HGLKLLTKNGHVYKYDGFRESEFEKLSDFFKTHYRLELME
consensus            61 . ...... *..... ...... ....*.......***.... ......*........ .


PBANKA_130530_prot  113 RKLATKGWNWGEFKLENSNLIFDIDKKYAFNINTNNINQLNVQIKTDIAIELKNDENKQN
PF14_0393_prot      111 RKIATKGWNWGEFKLENSNLCFDIDNKYAFNLPTNNINQLNVQIKTDIAMEFKNDENNNK
YML069W_prot        104 REHSLRGWNWGKTDLARNEMVFALNGKPTFEIPYARINNTNLTSKNEVGIEFNIQDEEYQ
Q08945_SSRP1_HUMAN  100 KDLCVKGWNWGTVKFGGQLLSFDIGDQPVFEIPLSNVSQCTTGKNEVTLEFHQNDD----
consensus           121 ......*****..........*... . .* .......................... ..


PBANKA_130530_prot  173 TNEDVLSEIRFYYPHENDENQN-----------------------------------FQD
PF14_0393_prot      171 GNEDFLAEIRFYYPHENDENQN-----------------------------------FQN
YML069W_prot        164 PAGDELVEMRFYIPGVIQTNVDENMTKKEESSNEVVPKKEDGAEGEDVQMAVEEKSMAEA
Q08945_SSRP1_HUMAN  156 -AEVSLMEVRFYVPPTQEDGVDP----------------------------------VEA
consensus           181   .. *.*.***.*......                                     . .


PBANKA_130530_prot  198 LKNNLLEKVNIGDSKSECIASLSNIPLLVPRGRYEIEMYSKTFKLHGKSYDFTVQYSNIN
PF14_0393_prot      196 LKNDLLEKVNIGDTKSESIASLSNIPLLVPRGRYDIEMYSSTFKLHGKSYDFNIQYTNIN
YML069W_prot        224 FYEELKEKADIGEVAGDAIVSFQDVFFTTPRGRYDIDIYKNSIRLRGKTYEYKLQHRQIQ
Q08945_SSRP1_HUMAN  181 FAQNVLSKADVIQATGDAICIFRELQCLTPRGRYDIRIYPTFLHLHGKTFDYKIPYTTVL
consensus           241  ......*  ..... ..*.. ...... *****.*..*.....*.**............


PBANKA_130530_prot  258 KMLLVPKTNSNQYILIFSLNNKIKQGQTEYPFILIQLSNDDDMDLDINASEEDIQN---Y
PF14_0393_prot      256 KMILVPKSNSNQYVLIFSLSNKMKQGQTEYPFILIQLNNDDDMELDISASDEVMTK---Y
YML069W_prot        284 RIVSLPKADDIHHLLVLAIEPPLRQGQTTYPFLVLQFQKDEETEVQLNLEDEDYEENYKD
Q08945_SSRP1_HUMAN  241 RLFLLPHKDQRQMFFVISLDPPIKQGQTRYHFLILLFSKDEDISLTLNMNEEEVEKRFEG
consensus           301 .....*.. ...........  ..****.*.*.... . *...........*.... . .


PBANKA_130530_prot  315 KLEKTLTGKAYDVVTRLFTALAKKNAIIPGDYRTAKNEHGITCSYRAASGQLYPLNKYFL
PF14_0393_prot      313 KLEKTISGKAHDVVTKLFTALVNKNVIVPGDYRTSKNQHGITCSYRAASGQLYPLNKYFL
YML069W_prot        344 KLKKQYDAKTHIVLSHVLKGLTDRRVIVPGEYKSKYDQCAVSCSFKANEGYLYPLDNAFF
Q08945_SSRP1_HUMAN  301 RLTKNMSGSLYEMVSRVMKALVNRKITVPGNFQGHSGAQCITCSYKASSGLLYPLERGFI
consensus           361 .*.*...... ....... .*.......**............**..*..*.****...*.


PBANKA_130530_prot  375 FVVKPVILISFDDIVTLSFQRT-GNINQHRFFSLIIKHKRG-ISYEYTNIDKSEYAPLLE
PF14_0393_prot      373 FIVKPVILISFDDIVTLSFQRT-GNINQHRFFSLIIKHKRG-MSYEYTNIDKSEYNPLLT
YML069W_prot        404 FLTKPTLYIPFSDVSMVNISRAGQTSTSSRTFDLEVVLRSNRGSTTFANISKEEQQLLEQ
Q08945_SSRP1_HUMAN  361 YVHKPPVHIRFDEISFVNFARG---TTTTRSFDFEIETKQG-TQYTFSSIEREEYGKLFD
consensus           421 ...**...*.*... .. ..*. ... ..*.* . ...... ... ...*.. *. .*..


PBANKA_130530_prot  433 FLKSKNLNIQDDANVSEKKTDFDDDDDDLSES--------DEEDYVAEEEDEDDDNDDDD
PF14_0393_prot      431 FLKSKNINIQDDANDLEKKQDFHNELDESDEEEYVADDDDDEEDYVAEEEDEDDDGDDDD
YML069W_prot        464 FLKSKNLRVKNE--DREVQERLQTALGSDSDEEDINMGSAGEDDESVDEDFQVSSDNDAD
Q08945_SSRP1_HUMAN  417 FVNAKKLNIKNRGLKEGMNPSYDEYADSDEDQHDAYLERMKEEGKIREENANDSSDDSGE
consensus           481 *...*....  .... ... ............... ..  .*......*....  .....


PBANKA_130530_prot  485 EYDDEDDDK---------------------------------------------------
PF14_0393_prot      491 DDEEEEEEEEEEDDDK--------------------------------------------
YML069W_prot        522 EVAEEFDSDAALSDAEGGSDEERPSKKPKVE-----------------------------
Q08945_SSRP1_HUMAN  477 ETDESFNPGEEEEDVAEEFDSNASASSSSNEGDSDRDEKKRKQLKKAKMAKDRKSRKKPV
consensus           541 . ............     .          .                             

PBANKA_130530_prot      ------------------------------------------------------------
PF14_0393_prot          ------------------------------------------------------------
YML069W_prot            ------------------------------------------------------------
Q08945_SSRP1_HUMAN  537 EVKKGKDPNAPKRPMSAYMLWLNASREKIKSDHPGISITDLSKKAGEIWKGMSKEKKEEW
consensus           601                                                             


PBANKA_130530_prot      ------------------------------------------------------------
PF14_0393_prot          ------------------------------------------------------------
YML069W_prot            ------------------------------------------------------------
Q08945_SSRP1_HUMAN  597 DRKAEDARRDYEKAMKEYEGGRGESSKRDKSKKKKKVKVKMEKKSTPSRGSSSKSSSRQL
consensus           661                                                             


PBANKA_130530_prot      -----------------------------------------------------
PF14_0393_prot          -----------------------------------------------------
YML069W_prot            -----------------------------------------------------
Q08945_SSRP1_HUMAN  657 SESFKSKEFVSSDESSSGENKSKKKRRRSEDSEEEELASTPPSSEDSASGSDE
consensus           721                                                      
