# Supplementary material for: Experimentally controlled downregulation of the histone chaperone FACT in Plasmodium berghei reveals that it is critical to male gamete fertility
Source: Cell Microbiol. 2011 Dec;13(12):1956–74. doi: 10.1111/j.1462-5822.2011.01683.x (PMC3429858; doi:10.1111/j.1462-5822.2011.01683.x)
Supplement: Supplementary file 4 [file cmi0013-1956-SD5.rtf]

                                                                                 

PbANKA_060190         1 -----MDGMKKFKDMKMGGKEVKKRRKNK-------KDPHAPKRSLSAYMFFAKEKRAEIIT 
PbANKA_071290         1 --------MATKTQKKVIKK---QNKKKK-------KDPLAPKRALSAYMFYVKDKRLEIIQ 
S.cerevisiae NHP 6B   1 --------MAATKEAKQPKEPKKRTTRRK-------KDPNAPKRRLSAYMFFANENRDIVRS 
S.cerevisiae NHP 6A   1 --------MVT------PREPKKRTTRKK-------KDPNAPKRALSAYMFFANENRDIVRS 
T.annulata            1 --------MAS-KVAKSAGK---KSKRAK-------KDPNAPKRALSSYMFFAKEKRAELVR 
K.lactis              1 --------MAA---------PRKKTQRKK-------KDPNAPKRALSAYMFFANENRDIVRA   
HumanSSRP1          506 NEGDSDRDEKKRKQLKKAKMAKDRKSRKKPVEVKKGKDPNAPKRPMSAYMLWLNASREKIKS 
                                                                                                 
                    
PbANKA_060190        51 RDPSLSKDVATVGKMIGEAWNKLDEREKAPYEKKAQEDKIRYEKEKMEYAKSKMK-- 
PbANKA_071290        45 ERPELAKEVAQVGKLIGEAWGQLTPAQKAPYEKKAELDKVRYSKEIEEYRKTKE--- 
S.cerevisiae NHP 6B  48 ENPDVT--FGQVGRILGERWKALTAEEKQPYESKAQADKKRYESEKELYNATRA--- 
S.cerevisiae NHP 6A  42 ENPDIT--FGQVGKKLGEKWKALTPEEKQPYEAKAQADKKRYESEKELYNATLA--- 
T.annulata           44 DNPDLARDVAAVGKLVGAAWNSLDESEKAPYEKLAEADRARYEKEKAAYNK------
K.lactis             39 ENPGIT--FGQVGRILGEKWKALNEDEKAPYEAKAEADKKRYESEKELYIATKAQSE 
Human SSRP1         568 DHPGIS--ITDLSKKAGEIWKGMSKEKKEEWDRKAEDARRDYEKAMKEYEGGRGESS 
              

Figure S4. Alignment of NHP6 of S. cerevisiae with the HMGB proteins of Plasmodium berghei and the C terminus of SSRP1 of H. sapiens
